# Fatigue following radiotherapy of low-risk early breast cancer – a randomized controlled trial of intraoperative electron radiotherapy versus standard hypofractionated whole-breast radiotherapy: the COSMOPOLITAN trial (NCT03838419)

**DOI:** 10.1186/s13014-020-01581-9

**Published:** 2020-06-01

**Authors:** Tobias Forster, Cornelia Jäkel, Sati Akbaba, David Krug, Robert Krempien, Matthias Uhl, Matthias Felix Häfner, Laila König, Stefan Alexander Koerber, Semi Harrabi, Denise Bernhardt, Rouven Behnisch, Johannes Krisam, Andre Hennigs, Christof Sohn, Jörg Heil, Jürgen Debus, Juliane Hörner-Rieber

**Affiliations:** 1 Department of Radiation Oncology, Heidelberg University Hospital, Heidelberg University, Im Neuenheimer Feld 400, 69120 Heidelberg, Germany; 2 Heidelberg Institute of Radiation Oncology (HIRO), Heidelberg, Germany; 3 National Center for Tumor Diseases (NCT), Heidelberg, Germany; 4 Clinical Cooperation Unit Radiation Oncology, German Cancer Research Center (DKFZ), Heidelberg, Germany; 5 Department of Radiation Oncology, University Hospital Schleswig Holstein, Kiel, Germany; 6 Department of Radiation Oncology, Helios Hospital Berlin-Buch, Berlin, Germany; 7 Institute of Medical Biometry and Informatics, Heidelberg University, Heidelberg, Germany; 8 Department of Gynecology and Obstetrics, Heidelberg University, 69115 Heidelberg, Germany; 9 Heidelberg Ion-Beam Therapy Center (HIT), Department of Radiation Oncology, Heidelberg University Hospital, Heidelberg, Germany; 10 German Cancer Consortium (DKTK), partner site Heidelberg, Heidelberg, Germany

**Keywords:** Fatigue, Radiotherapy, Breast cancer, Intraoperative electron radiotherapy, Whole-breast radiotherapy

## Abstract

**Background:**

Fatigue is one of the most common and distressing side-effects of breast cancer radiotherapy. According to current guidelines, accelerated partial breast irradiation (APBI) may be considered as an alternative treatment option for women with early-stage low-risk breast cancer. One method for APBI is single-dose intraoperative radiotherapy (IORT) applied directly to the tumor bed during breast conserving surgery (BCS). The COSMOPOLITAN trial therefore aims to analyze the intensity of fatigue following single-shot IORT with electrons (IOERT) compared to conventional hypofractionated whole breast irradiation (WBI) in low risk early breast cancer patients.

**Methods:**

This trial is conducted as a multicenter, prospective, randomized, two-arm phase II study comparing the intensity of fatigue in early-stage breast cancer (cT1cN0cM0, tumor size < 2,5 cm, ER pos. Her2neu neg., age > 50 years) treated either with WBI or APBI after BCS. Secondary outcomes investigated are tumor control, overall survival (OS), disease-free survival (DFS), acute and chronic toxicity, quality of life (QoL) and cosmesis. A total of 202 patients will be randomized into two arms: Patients in arm A will receive WBI (40.05 Gy, 15 fractions) after surgical resection, while patients in arm B will receive IOERT (21 Gy to the 90%-isodose) during BCS. Fatigue will be assessed 12 weeks post surgery with the help of the Functional Assessment of Chronic Illness Therapy (FACIT) Fatigue Scale.

**Discussion:**

The present trial aims to evaluate treatment response to compare single-shot intraoperative electron APBI to conventional WBI following BCS in early-stage low risk breast cancer patients. Fatigue is selected as the primary, patient-reported endpoint due its major clinical relevance.

**Trial registration:**

The study is prospectively registered on February 12th, 2019: Clinicaltrials.gov, NCT03838419. “Intraoperative Electron Radiotherapy for Low-risk Early Breast Cancer (COSMOPOLITAN)”.

**Study status:**

Ongoing study. Start of recruitment was December 2019.

## Background

Breast cancer is the most frequent cancerous disease in women [1, 2]. For patients with early stage breast cancer standard of care is breast conserving-surgery (BCS) followed by adjuvant radiotherapy and adjuvant systemic therapy according to the estrogen (ER) and progesterone receptor (PR) as well as human epidermal growth factor receptor 2 (HER2) status [1]. In several randomized controlled trials (RCTs) and meta-analyses, adjuvant whole-breast radiotherapy (WBI) after BCS has been shown to significantly decrease the risk of local (in-breast) recurrence and to improve breast-cancer mortality as well as OS [3, 4]. However, when selecting patients at a low a priori risk of local recurrence, only a small breast-cancer specific mortality benefit but no overall survival advantage was detected in the recent meta-analysis by the Early Breast Cancer Trialists Collaborative Group (EBCTCG) [4]. Consequently, several RCTs addressed the question whether the addition of adjuvant WBI to endocrine therapy improves the outcome of patients with early stage, low risk breast cancer [5–9]. Nevertheless, all of the individual trials detected a significant benefit for adjuvant WBI in terms of local control while no benefit for OS was found [5–9]. The effect of adjuvant WBI in addition to endocrine therapy was not limited to improved local control but also significantly improved DFS, as especially fewer axillary recurrences were reported in patients undergoing radiotherapy [6, 9]. This positive result was also confirmed after 10-year long term observation [10]. Thus, adjuvant WBI remains the standard of care after BCS in patients with early stage, low risk breast cancer.

As most of local recurrences occur in the proximity of the primary tumor bed, partial breast-irradiation delivered exclusively to the original tumor location has gained increased attention during the past couple of years [11–13]. Hereby, smaller target volumes allow for an increase in dose per fraction combined with a decrease in overall treatment time enabling accelerated partial breast irradiation (APBI). Several different techniques have been used to deliver APBI. Multicatheter brachytherapy has been one of the first techniques studied for APBI. 10-year results are available from a small single-center RCT and show comparable outcomes after WBI and APBI with multicatheter brachytherapy [14]. In 2016, 5-year results from a multi-center RCT conducted by the GEC-ESTRO have been published showing that APBI is non-inferior compared to WBI [15].

The probably most easily implementable approach for APBI is the application of percutaneous radiotherapy with a conventional linear accelerator as it is universally available. Besides some other studies, the IMPORT LOW-Trial compared partial-breast to whole-breast radiotherapy for low-risk early breast cancer using a standard external beam technique [16]. Five-year local recurrence rate were similar in all 3 arms and non-inferiority of the reduced dose-arm and the partial breast irradiation arm was shown [16]. Adverse events were comparable between the treatment arms with significantly fewer adverse effects in breast appearance and firmness in one or both of the experimental arms [16].

The ultimate form of APBI is single-dose IORT delivered exclusively to the tumor bed during BCS. Two large RCTs employing different techniques of IORT have been published so far: the ELIOT and the TARGIT-A-trial [17, 18]. Although, both trials presented promising results, significantly more local recurrences were detected in the IORT arms compared to standard WBI due to different shortcomings: While in the ELIOT-trial the inclusion criteria were non-restrictive resulting in the enrollment of a considerable number of patients with higher risk factors [18], the TARGIT-A trial also allowed the inclusion of patients who received IORT during a second operative procedure (post-pathology stratum), which impeded non-inferiority of results [18]. A meta-analysis of 5 RCTs comparing APBI to WBI was conducted by the TARGIT A-investigators [19]. While breast cancer-related mortality was similar, APBI resulted in a significant decrease of non-breast cancer-related mortality and a borderline significant improvement in OS when using a random-effects model [19]. Based on the above mentioned studies, both national guidelines in Germany, but also international guidelines suggest the use of APBI in patients with early stage, low risk breast cancer [1, 2, 20, 21]. Nevertheless, most guidelines recommend the application of APBI preferably within a clinical trial [1, 21].

Fatigue is one of the most common and distressing side-effects reported by breast cancer patients and occurs in more than 80% of patients during radiotherapy [22–27]. During the course of radiotherapy, fatigue usually increases, but typically subsides within weeks after the end of radiation treatment [24, 28]. However, in up to 40% of cases, it can persist long after the completion of therapy [29–31]. Fatigue is usually described as a general physical or mental exhaustion that negatively affects QoL due to functional disability and psychological distress [28, 32]. Psychosocial problems like fatigue, depression or cognitive limitations in occupationally active cancer survivors are distinctly associated with problems at work [33]. The potential economic impact of fatigue is considerably high: 75% of patients and 40% of caregivers are forced to change their employment status due to cancer-related fatigue [34, 35]. A recent study about employment participation in early-breast cancer patients further highlighted the enormous socioeconomic impact of fatigue by showing that fatigue patients were more likely to experience diminished employment after 2 years of follow-up [36].

Previous analyses reported that the level of fatigue rises with cumulative radiation dose and might be related to the duration of treatment [37, 38]. Both, total doses as well as field sizes are known to influence severity of fatigue [38–40]. For APBI the target volume only includes the former tumor cavity and not the whole breast tissue and total treatment time is usually reduced compared to WBI. Hence, early stage breast cancer patients treated with APBI instead of WBI are expected to recover faster from cancer-related fatigue leading to improved QoL compared to patients receiving WBI.

The aim of the present trial is to compare single-shot intraoperative APBI with electrons to standard hypofractionated WBI for patients with low-risk early stage breast cancer. Due to its high socio-economic and clinical impact fatigue is chosen as the primary, patient-reported endpoint for this study.

## Methods/design

### Trial aim

The purpose of this trial is to analyze intensity of fatigue in early-stage breast cancer treated with WBI or APBI after BCS. We propose that patients treated with APBI have lower fatigue levels after radiotherapy based on the FACIT Fatigue Assessment Questionnaire compared with patients treated with standard WBI.

### Trial design

**Fig. 1:**
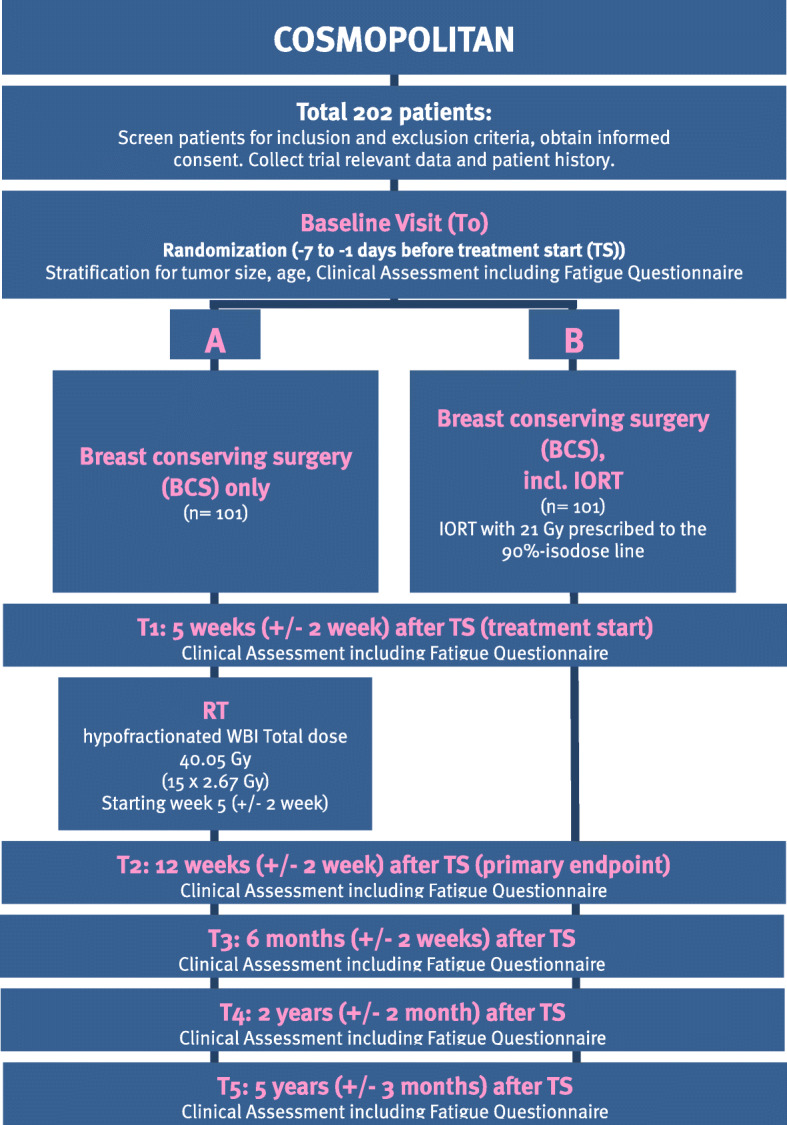
Trial flow-chart

The COSMOPOLITAN trial is a multicenter, prospective, randomized, two-arm phase II study. The trial has been designed by the study initiators at the Department of Radiation Oncology of the University of Heidelberg. The trial is carried out at the University of Heidelberg, Department of Radiation Oncology and at the Helios Hospital Berlin-Buch, Department of Radiation Oncology. The inclusion of further study sites is planned. A list of study sites can be obtained from the corresponding author on reasonable request. The University of Heidelberg is responsible for trial management and coordination, as well as quality assurance including reporting, monitoring and database management. The current version of the study protocol is version 1.1 from January, 24th 2019 (supplementary material 1). The study workflow and treatment arms are depicted in Fig. 1. Two-hundred-and-two women with early stage, low risk breast cancer eligible for breast-conserving surgery fulfilling the inclusion criteria will be enrolled in this phase II clinical trial.

#### Inclusion criteria

Patients meeting the following criteria will be included in the trial:

- Histologically confirmed invasive breast cancer
- Total tumor size < 2.5 cm
- cN0
- Estrogen receptor positive, HER2-negative on immunohistochemistry
- Age ≥ 50 years
- ECOG Performance status ≤2
- Ability of subject to understand character and individual consequences of the clinical trial
- Written informed consent (must be available before enrolment in the trial)

#### Exclusion criteria

Patients presenting with one of the following criteria will not be included in the trial:

- G3
- Extensive microcalcifications
- Invasive lobular carcinoma
- Clinically involved lymph nodes
- No invasive axillary lymph node staging planned
- Patients with significant mental or physical comorbidities that preclude regular follow-up
- Neoadjuvant chemotherapy or neoadjuvant endocrine therapy
- Previous radiotherapy of the breast
- Known carcinoma < 5 years ago (excluding Carcinoma in situ of the cervix, basal cell carcinoma, squamous cell carcinoma of the skin) requiring immediate treatment interfering with study therapy
- Pregnant or lactating women
- Participation in another competing clinical study or observation period of competing trials

Patients are withdrawn from the study, if a R1/2 resection status is diagnosed postoperatively. In this case, re-resection and additional whole-breast irradiation should be taken into account.

### Randomization

After meeting eligibility criteria, 202 patients will be randomly assigned to intraoperative APBI or WBI. Appropriate patients will be allocated in a concealed fashion in a 1:1 ratio by means of randomization using a centralized web-based tool (www.randomizer.at), to achieve comparable intervention groups. Randomization will be stratified with respect to invasive tumor size (≤ 1 cm^3^, > 1 cm^3^) and age (≤ 60 years, > 60 years). Block randomization with varying block lengths will be performed to achieve in total equal group sizes.

### Study treatment

#### Arm A - conventional arm

WBI with a total dose to the breast of 40.05 Gy in 15 fractions is administered after full recovery from surgical resection.

#### Arm B - experimental arm

During BCS, patients will receive IOERT with a total dose of 21 Gy prescribed to the 90%-isodose.

### Trial objectives

The primary objective is the assessment of fatigue from baseline (before treatment start) compared to 12 weeks after treatment start as assessed by the FACIT Fatigue Assessment Questionnaire. Secondary outcomes investigated are local tumor control in the index quadrant of the breast and in the ipsilateral breast (time from randomization until local tumor progression, death without prior local progression, or end of follow-up at the respective site), regional tumor control (time from randomization until occurrence of regional lymph node metastases, death without prior regional tumor progression, or end of follow-up), distant tumor control (time from randomization until occurrence of distant metastases, death without prior distant progression, or end of follow-up), OS (time from randomization until death or end of follow-up), DFS (time from randomization until the first occurrence of local recurrence, regional lymph node metastases, distant metastases, tumor-related death, death without prior progression, or end of follow-up), secondary malignancies, acute and chronic toxicity, QoL and cosmesis. Toxicity, cosmesis and QoL assessment are performed according to international validated scores and questionaires (Table 2). Fatigue at 5 weeks after treatment start (T1) will be evaluated in both study groups as secondary endpoint to assess the potential influence of IORT on fatigue. Additionally, the Prosigna/PAM50-assay will be applied to further evaluate the potential prognostic impact of tumor biology and gene expression-analysis in supporting adequate patient selection.

### Treatment planning and radiation therapy

#### Arm A

Radiotherapy is administered after full recovery from surgical resection, usually after 5 weeks of BCS. Patients will receive computed tomography-based 3-dimensional treatment planning. The total dose to the breast is 40.05 Gy in 15 fractions. No tumor bed boost or regional nodal irradiation shall be delivered.

Target contouring of the CTV-breast is performed according to the ESTRO-guidelines [41, 42]. An additional margin for inter- and intrafractional positioning uncertainties of 5–10 mm is added to generate the PTV-breast. The PTV-breast_EVAL (generated by subtraction of the 3 mm below the skin from the PTV-breast) should receive 95–107% of the prescribed dose. Dose constraints of normal tissue will be respected according to Table 1.

**Table 1:** Dose constraints of normal tissue

Organ at risk	Dose constraints
**Heart**	Dmean < 3 Gy, Dmax < 30 Gy (minor violation < 40 Gy)
**Ipsilateral Lung**	Dmean < 8 Gy, V20Gy < 10% (minor violation < 15%)
**Contralateral Breast**	Dmean < 3 Gy, Dmax < 20 Gy
**Spinal cord**	Dmax < 45 Gy

Physical Quality assurance (QA) is not defined in the protocol, but is done according to standard operating procedures of the resepctive study centers. At Heidelberg University, for the standard arm of the COSMOPOLITAN trial, both 3D-conformal radiotherapy (3-D-CRT) and intensity-modulated radiotherapy (IMRT) are used. For QA, secondary dose calculations are performed with a Monte Carlo algorithm for the IMRT plans, while simpler algorithms are applied for the 3-D-CRT plans. IMRT plans are intermittently measured.

#### Arm B

Patients will receive IOERT with a total dose of 21 Gy prescribed to the 90%-isodose. After resection of the tumor, a lead or aluminum shield will be inserted between the back side of the breast tissue and the anterior part of the major pectoralis muscle to protect the thoracic wall. The resection margins are sutured over the shield according to the instructions by Veronesi et al. [43]. An appropriate size and shape of the tube will be selected, taking into account the tumor size, the size of the treated breast and the potential effect on cosmesis. However, the minimum tube size shall be at least 4 cm, in order to create a 2 cm safety margin around the sutured resection margins of the tumor bed. The disk diameter of the shield will be at least 1.5 cm larger than the chosen tube size. The linear accelerator delivers electrons at variable energies (6, 9 and 12 MeV). Depending on the target thickness the adequate electron energy is chosen.

### Follow up

The baseline Visit will be performed after enrolment of the patient into the study and will be scheduled − 7 to − 1 day before planned treatment start. During the baseline visit (T0) a clinical assessment as well as analysis of QoL including fatigue is scheduled. Patients will also receive photographic documentation in standardized patient positions for cosmetic assessment: Two frontal photographs (one with the arms raised above the head and the other with both arms alongside the body) and one profile photograph of the treated breast (arms raised) will be taken.

Both study groups will be evaluated after BCS at 5 weeks after treatment start (T1), to assess the potential influence of IORT on fatigue (secondary endpoint). In group A this time point corresponds to 5 weeks after BCS, just before the start of RT; in group B this corresponds to 5 weeks after BCS incl. IORT.

The second, third, fourth and fifth study visits (T2-T5) are planned 12 weeks (primary endpoint), 6 months, 2 and 5 years (secondary endpoints) after the treatment start. These visits will include a clinical assessment as well as analysis of QoL including fatigue (EORTC and Fatigue Assessment Questionnaires, BREAST-Q and BCTOS, see below for details). Photographic assessment of cosmetic results is also planned except for the last visit after 5 years. The follow-up workflow is depicted in Table 2.

**Table 2:** Follow-up workflow

	T0 Baseline	T1 5 weeks	T2 12 weeks	T3 6 months	T4 2 years	T5 5 years
**Medical history**	X	X	X	X	X	X
**Fatigue Assessment (FACIT Fatigue Scale)**	X	X	X	X	X	X
**EORTC QLQ-C30**	X	X	X	X	X	X
**BREAST-Q and BCTOS**	X	X	X		X	
**Documentation of medication**	X	X	X	X	X	X
**Documentation of AEs**		X	X			
**Photographic documentation of cosmetic assessment**	X	X	X		X	

### Outcome measures

#### Primary endpoint: fatigue testing

The primary endpoint is change of fatigue after APBI or WBI from baseline to 12 weeks after treatment start (T2 vs. T0). Fatigue will be assessed with the help of the Functional Assessment of Chronic Illness Therapy (FACIT) Fatigue Scale, which consists of a 13-item multidimensional self-assessment form evaluating quantity of fatigue and distress [44]. The FACIT Fatigue Scale is extensively applied in cancer patients [45, 46]. Using the FACIT Fatigue Scale questionnaire, patients are asked to indicate how frequent each item was for them “during the past 7 days” applying a 5-point scale (“not at all” to “very much”). The range of possible scores is 0–52, with 0 being the worst possible score and 52 the best.

#### Secondary endpoints

Secondary analysis include local tumor control, regional and distant recurrence rates, disease-free as well as overall survival, assessment of acute and chronic toxicity (CTCAE v5.0), QoL (EORTC QLQ-C30, BREAST-Q BCS [47, 48], BCTOS-12 [49]) and cosmesis. Cosmesis will be evaluated according to the proposed method by Vrieling et al. [50]. Additionally, there will be a cosmesis assessment by both the treating physician and the patient. Furthermore, gene expression analysis using the Prosigna/PAM50-assay will be performed on the operatively resected tumor tissue as a secondary endpoint to evaluate the potential prognostic/predictive impact on choice of radiotherapy modality [51, 52].

The BREAST-Q questionnaire is a validated survey instrument especially developed for patients undergoing breast surgery. An extra BREAST-Q BCS module exists, which is specifically designed for patients undergoing BCS measuring QoL and satisfaction [47, 48]. Both of these topics are divided into three subscales i.e., QOL: physical, psychosocial and sexual well-being; Satisfaction: satisfaction with cosmetic outcome (breast appearance), satisfaction with overall outcome and satisfaction with care. Patients are asked to rate each item question on a four-point scale. The BREAST-Q is separated into a pre- and post-surgery version.

The Breast Cancer Treatment outcome scale (BCTOS-12) contains 12 items, which are assigned to two internally consistent subscales: [49] 1) Functional Status (e.g. change in shoulder mobility etc.), 2) Aesthetic Status (e.g. shape of the breast etc.). Patients are instructed to rate each item of the BCTOS-12 on a four-point scale evaluating the differences between the treated and the untreated breast (1 = no difference, 4 = large difference). The score for each subscale is the mean of the ratings over all items belonging to that subscale. A higher score reflects a poorer status (i.e. a larger difference between the treated and the untreated breast).

### Statistical analysis

#### Hypotheses

The null hypothesis to be tested states that the change in the FACIT-Fatigue score, between baseline and week 12 after treatment start is equal for both groups. This hypothesis will be tested at a two-sided level of significance of α= 0.05 against the alternative hypothesis.

#### Sample size calculation

The sample size calculation is based on the primary endpoint change of fatigue from baseline to week 12 after Treatment start (study visit T2) between the groups APBI and WBI measured via the FACIT-Fatigue score. An improvement in 6 of 13 items by one point is considered clinically relevant resulting in a clinically relevant effect size of 6 points. Assuming a standard deviation of 12 [53] and with a type I error rate of α = 0.05 (two-sided), a two sample t-test requires a total sample size of *n* = 172 patients (86 per group) to achieve a power of 1 − β = 0.90 for revealing an effect of 6 points. Adjusting for the covariates baseline fatigue, age, and tumor size in a linear model is assumed to yield less unexplained variance and thus to an additionally increased power. Taking a dropout rate of 15% percent into account, *n* = 202 patients need to be randomized. Sample size calculation was carried out using ADDPLAN v 6.1.

#### Analysis methods

The full analysis set (FAS) includes all randomized patients and they will be analyzed according to the intention-to-treat (ITT) principle. In addition to the evaluation of the FAS, a per-protocol population (PP) analysis will be performed as a sensitivity analysis. The hypothesis test will be conducted using a linear model with the dependent variable “change in FACT-F score between baseline and week 12 after treatment start” and the independent factors treatment group (A/B), baseline FACT-F score, tumor size, and age. The effect will be calculated alongside a 95% confidence interval. Missing data for the primary outcome variable will be replaced using multiple imputation [54] taking the covariates treatment group, baseline FACT-F score, tumor size, and age into account by application of the fully conditional specification method [55]. In addition to the evaluation of the FAS, a PP analysis will be performed as a sensitivity analysis. The secondary outcomes will be analyzed descriptively by tabulation of the measures of the empirical distributions. According to the scale level of the variables, means, standard deviations, medians, 1st and 3rd quartiles as well as minimum and maximum or absolute and relative frequencies, respectively, will be reported. Further, for the secondary endpoints OS, DFS, local, regional, and distant tumor control, a Kaplan-Meier analysis will be performed and a between-groups comparison via a descriptive log-rank test will be conducted. Adverse and Serious Adverse Events will be tabulated and absolute and relative frequencies with 95% confidence intervals will be calculated. The severity and the relationship to the treatment will be given. Possible differences between the treatment groups will be tested using the chi-squared test. Descriptive *p*-values of the corresponding statistical tests comparing the treatment groups and associated 95% confidence intervals will be given. Further exploratory analyses will be performed to identify subgroups and potential moderator variables of patients profiting distinctly from the investigated interventions. Analyses will be conducted using SAS v9.4 (SAS Institute, Cary, NC).

## Discussion

As studies investigating APBI are still scarce, current international and German guidelines suggest the use of APBI for low-risk early stage breast cancer patients but recommend the application of APBI preferably within a clinical trial [1, 2, 21]. Two recent RCTs analyzed the role of intraoperative single-shot APBI for early-stage low-risk breast cancer patients: the ELIOT and the TARGIT-A-trial [17, 18]. However, the results of both studies were impaired by limitations in selecting adequate patients.

On the one hand, the TARGIT-A trial compared single-shot 20 Gy of 50 kV-IORT with 50 Gy of WBI in 25 fractions and reported an increased 5-year local recurrence rate of 3.3% for the IORT-arm vs. 1.3% for WBI-arm, which was still acceptable within the predefined threshold level (*p* = 0.042). However, the borderline increased local recurrence rate for the IORT-arm was attributed to the inclusion of patients in whom IORT was delivered as a delayed second procedure by reopening the lumpectomy cavity (postpathology stratum). While local recurrence was 2.1% with IORT and 1.1% with WBI in the prepathology stratum (*p* = 0.31), the local recurrence rate rose to 5.4% for IORT and 1.7% for WBI in the postpathology stratum (*p* = 0.069), which was higher than the pre-specified non-inferiority margin of 2·5% at 5 years. This particularly important result underlines the need for concomitant delivery of intraoperative radiotherapy and lumpectomy [56].

On the other hand, the ELIOT-trial compared one dose of 21 Gy of IOERT to WBI with 50 Gy and a tumor bed boost over 6 weeks and described an increased 5-year local recurrence rate of 4.4% for patients receiving IORT and 0.4% for patients treated with WBI (*p* = 0·0001), which was still below the pre-specified equivalence margin of 7.5% [18]. However, the trial also included less suitable patients with risk factors such as tumor size > 2 cm, tumor grade G3, positive nodes, and triple-negative tumors. After excluding these patients with risk factors, the 5-year local recurrence rate decreased to only 1.5% in the IORT-arm. Based on the above mentioned results, inclusion criteria will be highly restrictive in the COSMOPOLITAN trial. The ASTRO has recently published selection criteria for APBI, which will be used for guaranteeing that only low-risk early-breast cancer patients will be treated with APBI in the current trial [20].

Due to its considerably high socio-economic and clinical impact fatigue is chosen as the primary, patient-reported endpoint for this study [34, 35]. Few first and mainly retrospective studies with small patient numbers reported lower levels of acute fatigue in breast cancer patients receiving APBI instead of WBI [40, 45, 57]. Perez et al. retrospectively evaluated side-effects and QoL following WBI vs. APBI for early-stage low-risk cancer and described significantly reduced severity of fatigue besides other factors after APBI compared to WBI [57]. In a matched-pair analysis, Taunk et al. retrospectively compared radiation-induced fatigue in patients treated with APBI or hypofractionated WBI to patients receiving normofractionated WBI [40]. During radiotherapy and the first follow-up visit, significantly lower fatigue levels were detected for patients in the APBI group [40]. A pilot study by Albuquerque et al. evaluated the impact of partial versus whole breast irradiation on fatigue, perceived stress, QoL and natural killer cell activity in 30 women with breast cancer and supported the above mentioned findings by reporting that partial breast irradiation results in more rapid recovery from cancer-related fatigue compared to WBI [45]. Furthermore, the GEC-ESTRO study group recently investigated QoL following APBI with interstitial brachytherapy and described significantly reduced fatigue after APBI compared to WBI [58].

The primary goal of the COSMOPOLITAN study is to show that BCS followed by APBI with electrons results in a significantly better tolerability of adjuvant radiotherapy in terms of fatigue compared to BCS followed by hypofractionated WBRT. Fatigue is a major side effect of cancer treatments and is a main contributor to long-term quality of life-impairment as well as unemployment [32, 34–36]. The prognosis of women with early stage, low risk breast cancer is excellent and the risk of local recurrence after BCS has been shown to be in the range of 1–2% at 5 years after diagnosis [59]. Thus, maintaining a high QoL and minimizing morbidity and mortality of adjuvant treatment is crucial.

## Supplementary information

**Additional file 1.** Supplementary material 1: Current version of the study protocol, version 1.1 from January, 24th 2019.

## Data Availability

The data is collected, managed and processed electronically in the in-house research database. To ensure data quality and consistency, internal quality control measures will be carried out. The originals of all central study documents are kept at the Study Center for at least 15 years after the final report has been prepared. The dataset used and analyzed during the current study will be available from the corresponding author on reasonable request. Regulatory authorities may request access to all source documents, CRF and other trial documentation.

## Abbreviations

3-D-CRT: 3D-conformal radiotherapy
AE: Adverse Events
AMG: German Drug Law (Deutsches Arzneimittelgesetz)
APBI: Accelerated partial breast irradiation
ASTRO: American Society for Therapeutic Radiology and Oncology
BCS: Breast conserving-surgery
BDSG: Bundesdatenschutzgesetz
BRA: Breast retraction assessment
CHT: Chemotherapy
CRF: Case Report Form
CT: Computer tomography
CTCAE: Common Toxicity Criteria for Adverse Events
CTV: Clinical target volume
DFS: Disease-free survival
DSGVO: Datenschutzgrundverordnung, General Data Protection Regulation
DVH: Dose volume histogram
EC: Ethics Committee
ER: Estrogen receptor
FDA: US Food and Drug Administration
FSI: First Subject In
GCP: Good Clinical Practice
Gy: Gray
HIRO: Heidelberger Institut für Radioonkologie
IMRT: Intensity-modulated radiotherapy
IOERT: Intraoperative electron radiotherapy
IORT: Intraoperative radiotherapy
ISF: Investigator Site File
ISRCTN: International Standard Randomised Controlled Trial Number
IT: Information technology
ITT: Intention-to-treat
KPS: Karnofsky Performance Score
LPI: Last Patient In
LPO: Last Patient Out
OS: Overall survival
PI: Principle investigator
pBRA: Percentage BRA
PP: Per protocol
PR: Progesterone receptor
PTV: Planning target volume
QA: Quality assurance
QoL: Quality of Life
RCT: Randomized controlled trials
Ref: Reference length
RT: Radiation therapy
RTOG: Radiation Therapy Oncology Group
SAE: Severe adverse events
SOP: Standard operating procedures
TS: Treatment start
WBI: Whole-breast radiotherapy
