# Supplementary material for: Fatigue following radiotherapy of low-risk early breast cancer – a randomized controlled trial of intraoperative electron radiotherapy versus standard hypofractionated whole-breast radiotherapy: the COSMOPOLITAN trial (NCT03838419)
Source: Radiat Oncol. 2020 Jun 1;15:134. doi: 10.1186/s13014-020-01581-9 (PMC7268450; doi:10.1186/s13014-020-01581-9)
Supplement: Supplementary file 1 — Additional file 1. Supplementary material 1: Current version of the study protocol, version 1.1 from January, 24th 2019. [file 13014_2020_1581_MOESM1_ESM.pdf]

UNIVERSITÄTS
KLINIKUM
HEIDELBERG

RadioOnkologie und Strahlentherapie | Universitätsklinikum Heidelberg|
Im Neuenheimer Feld400| 69120 Heidelberg

CLINICAL TRIAL PROTOCOL

Intraoperative electron radiotherapy for low-risk early breast cancer

- COSMOPOLITAN -

financially supported by IntraOp

Randomized controlled Phase II trial

Version 1.1, 24.01.2019

Protokollcode: RADONK-COSMOPOLITAN-2018

Reg.-Nr.: NCT03838419

Principal Investigator: Prof. Dr. Dr. Jürgen Debus

Study Coordinator/ Co-Investigator: Dr. Juliane Hörner-Rieber

Universitätsklinikum Heidelberg
Klinik für RadioOnkologie und Strahlentherapie (Czerny-Klinik)
Im Neuenheimer Feld 400
69120 Heidelberg

CONFIDENTIAL: This protocol contains confidential information and is intended solely for the guidance of the clinical investigation. This protocol may not be disclosed to parties not associated with the clinical investigation or used for any purpose without the prior written consent of the Principal Investigator/ Coordinating Investigator.

UK
HD

Responsibilities

**Principal Investigator (PI):**	**Prof. Dr. Dr. Jürgen Debus**<br>Department of Radiation Oncology<br>University of Heidelberg<br>Im Neuenheimer Feld 400<br>69120 Heidelberg, Germany<br>Phone: +49 6221-56-8202<br>Fax: +49 6221-56-5353<br>E-Mail: juergen.debus@med.uni-heidelberg.de
**Study coordinator/Co-PI**	**Dr. Juliane Hörner-Rieber**<br>Department of Radiation Oncology<br>University of Heidelberg<br>Im Neuenheimer Feld 400<br>69120 Heidelberg, Germany<br>Phone: +49 6221-56-8202<br>Fax: +49 6221-56-5353<br>E-Mail: juliane.hoerner-rieber@med.uni-heidelberg.de
**Co-Investigator**	**PD Dr. Matthias Uhl**<br>Department of Radiation Oncology<br>University of Heidelberg<br>Im Neuenheimer Feld 400<br>69120 Heidelberg, Germany<br>Phone: +49 6221-56-8202<br>Fax: +49 6221-56-5353<br>E-Mail: matthias.uhl@med.uni-heidelberg.de
**Co-Investigator**	**Dr. Matthias Häfner**<br>Department of Radiation Oncology<br>University of Heidelberg<br>Im Neuenheimer Feld 400<br>69120 Heidelberg, Germany<br>Phone: +49 6221-56-8202<br>Fax: +49 6221-56-5353<br>E-Mail: matthias.haefner@med.uni-heidelberg.de
**Co-Investigator**	**Prof. Dr. Jörg Heil**<br>Department of Gynecology<br>University of Heidelberg<br>Im Neuenheimer Feld 440<br>69120 Heidelberg, Germany<br>Phone: +49 6221-56-7901<br>Fax: +49 6221-56-4328<br>E-Mail: joerg.heil@med.uni-heidelberg.de
**Co-Investigator**	**Dr. Tobias Forster**<br>Department of Radiation Oncology<br>University of Heidelberg<br>Im Neuenheimer Feld 400<br>69120 Heidelberg, Germany<br>Phone: +49 6221-56-8202<br>Fax: +49 6221-56-5353<br>E-Mail: tobias.forster@med.uni-heidelberg.de

Head of Clinical Trial Office RadioOncology:

Dr. Cornelia Jäkel
Department of Radiation Oncology
University of Heidelberg
Im Neuenheimer Feld 400
69120 Heidelberg, Germany
Phone: +49 6221-56-34093
Fax: +49 6221-56-8968
E-Mail: cornelia.jaekel@med.uni-heidelberg.de

Study assistance and documentation:

Clinical Trial Office RadioOncology
Department of Radiation Oncology
University of Heidelberg
Im Neuenheimer Feld 400
69120 Heidelberg, Germany
Phone: +49 6221-56-38613/-37748/-36318/-32682
Fax: +49 6221-56-1979
E-Mail: studien.radonk@med.uni-heidelberg.de

Trial statistican:

Dr. Johannes Krisam
Institute of Medical Biometry and Informatics,
University of Heidelberg
Im Neuenheimer Feld 130.3
69120 Heidelberg, Germany
Phone: +49 6221-56-34516
E-mail: krisam@imbi.uni-heidelberg.de

IT:

Nina Bougatf
Institute of Medical Biometry and Informatics,
University of Heidelberg
Im Neuenheimer Feld 130.3
69120 Heidelberg, Germany
Tel.: +49 6221-56-37656
Fax: +49 6221-56-8968
E-Mail: nina.bougatf@med.uni-heidelberg.de

Summary

Breast cancer is the most frequent malignancy in women[1, 2]. Standard therapy for early-stage breast cancer is breast conserving surgery (BCS) followed by adjuvant whole breast irradiation (WBI), which usually requires a treatment time of 3-6 weeks[1, 2]. Several randomized controlled trials (RCTs) demonstrated that postoperative WBI after BCS significantly decreased the risk of local (in-breast) recurrence and improved breast cancer mortality as well as survival[3, 4]. However, one of the most common side-effects of radiotherapy is fatigue, which is reported in up to 80% of cancer patients during treatment[5-10]. Especially in early-stage breast cancer patients it might be the only serious side-effect following adjuvant irradiation, as fatigue often significantly reduces quality of life due to resulting functional impairment and psychological distress[11, 12]. Fatigue additionally has a distinct socioeconomic impact: 75% of patients and 40% of caregivers are forced to change their employment status due to cancer-related fatigue[13, 14].

For reducing treatment-related toxicity, several RCTs consequently addressed the question whether adjuvant WBI could be omitted in early-stage, low risk breast cancer patients treated with endocrine therapy[15-20]. However, all these trials detected up to seven-time increased local recurrence rates without WBI following BCS[15-19]. Both, longer duration of radiotherapy and larger radiation field sizes are known to be associated with increase in treatment-related fatigue[21, 22]. Accelerated partial breast irradiation (APBI), delivered exclusively to the original tumor location and not to the surrounding breast tissue, might therefore be an alternative treatment option with fewer side-effects for early-stage, low risk breast cancer patients. Few previous trials have already reported comparable outcomes for highly selected low-risk breast cancer patients for APBI compared to conventional WBI[23-26]. First results also point out that APBI compared to WBI might be associated with less severity and intensity of fatigue[22, 27, 28].

One method for APBI is single-dose intraoperative radiotherapy (IORT) delivered directly to the tumor after resection. APBI with electron IORT might even contribute to further reducing treatment-related fatigue, as duration of adjuvant radiotherapy is dramatically shortened to one day compared to 3-5 weeks for conventional WBI. Data is still limited for APBI, hence current international and German guidelines suggest the use of APBI for low-risk early stage breast cancer patients but recommend the application of APBI preferably within a clinical trial[1, 2, 29].

The present trial therefore aims to evaluate treatment response to intraoperative electron APBI compared to conventional WBI following BCS in early-stage low risk breast cancer patients. The primarily investigated endpoint is the intensity of fatigue, as objectively assessed with the help of the Functional Assessment of Chronic Illness Therapy (FACIT) Fatigue Scale at 12 weeks after treatment start. Secondary endpoints include local, regional and distant tumor control, disease-free and overall survival, quality of life, cosmesis and toxicity. Additionally, the Prosigna/PAM50-assay will be used to further evaluate the potential prognostic impact of tumor biology and gene expression-analysis in supporting adequate patient selection for APBI.

Zusammenfassung

Das Mammakarzinom stellt die häufigste Krebserkrankung der Frau dar[1, 2]. Standardtherapie in frühen Stadien ist die brusterhaltende Operation (BCS) gefolgt von einer adjuvanten Ganzbrustbestrahlung (WBI), welche sich normalerweise über 3-6 Wochen erstreckt[1, 2]. Mehrere randomisierte, kontrollierte Studien (RCTs) zeigten, dass die postoperative WBI das lokale (in der Brust) Rezidivrisiko signifikant reduziert sowie die brustkrebsspezifische Mortalität und das Überleben verbessert[3, 4]. Jedoch ist eine der häufigsten Nebenwirkungen der Strahlentherapie eine Fatigue, von welcher bis zu 80% der Tumorpatienten während der Behandlung berichten[5-10]. Vor allem für Brustkrebspatientinnen im frühen Stadium kann sie nach Bestrahlung die einzige ernsthafte Nebenwirkung darstellen, da Fatigue oft zu einer signifikanten Reduktion der Lebensqualität aufgrund von funktionellen Beeinträchtigungen und psychischen Belastungen führt[11, 12]. Fatigue hat zusätzlich eine ausgeprägte sozioökonomische Bedeutung: 75% der Patienten und 40% der pflegenden Angehörigen sehen sich gezwungen, ihren Beschäftigungsstatus aufgrund von tumorbedingter Fatigue zu ändern[13, 14].

Um therapiebedingte Nebenwirkungen zu reduzieren, haben daher konsequenterweise einige RCTs die Frage evaluiert, ob Brustkrebspatientinnen im frühen Niedrigrisikostadium eine adjuvante Bestrahlung zusätzlich zur endokrinen Therapie überhaupt benötigen[15-20]. Jedoch wiesen alle diese Studien eine bis zu siebenfach erhöhte Lokalrezidivrate ohne WBI nach BCS auf[15-19]. Es ist bekannt, dass es zu einer Zunahme der therapiebedingten Fatigue bei längerer Bestrahlungsdauer und größerer Bestrahlungsfeldgröße kommt[21, 22].

Die akzelerierte Teilbrustbestrahlung (APBI), bei welcher nur die ursprüngliche Tumorlokalisation und nicht das umgebende Brustdrüsengewebe bestrahlt werden, könnte daher eine nebenwirkungsärmere Behandlungsalternative für Brustkrebspatientinnen in frühen Niedrigrisikostadien darstellen. Wenige bisherige Studien berichteten bereits von entsprechenden onkologischen Resultaten nach APBI im Vergleich zur konventionellen WBI für Brustkrebspatientinnen in frühen Niedrigrisikostadien[23-26]. Erste Ergebnisse weisen ebenfalls darauf hin, dass die APBI im Vergleich zur WBI mit einer geringeren Intensität und Schwere an Fatigue einhergeht[22, 27, 28].

Eine Technik, die APBI durchzuführen, ist die einmalige intraoperativen Bestrahlung (IORT), welche im Anschluss an die Resektion direkt auf das Tumorbett verabreicht wird. Die APBI als IORT mit Elektronen könnte sogar zur einer weiteren Reduktion der therapiebedingten Fatigue führen, weil die Dauer der adjuvanten Bestrahlung dramatisch auf einen Tag im Vergleich zu 3-5 Wochen für die konventionelle WBI verkürzt wird. Da die Datenlage zur APBI immer noch begrenzt ist, erlauben die aktuellen internationalen und deutschen Leitlinien die APBI für Brustkrebspatientinnen in frühen Niedrigrisikostadien, empfehlen aber bevorzugt die Anwendung innerhalb einer klinischen Studie [1, 2, 29].

Das Ziel der aktuellen Studie ist es daher, das Behandlungsansprechen auf die intraoperative APBI mit Elektronen im Vergleich zur konventionellen WBI bei Brustkrebspatientinnen in frühen Niedrigrisikostadien zu evaluieren. Der primär untersuchte Endpunkt ist die Intensität der Fatigue entsprechend der Testergebnisse der Functional Assessment of Chronic Illness Therapy (FACIT) Fatigue Skala 12 Wochen nach Behandlungsbeginn. Sekundäre Endpunkte sind die lokale, regional und distante Tumorkontrolle sowie das krankheitsfreie Überleben und das Gesamtüberleben, die Lebensqualität, Kosmetik und Toxizität. Zusätzlich wird der Prosigna/PAM50-Assay verwendet, um den möglichen prognostischen Einfluss der Tumorbiologie und Genexpressionsanalyse zur Unterstützung der Selektion geeigneter Patienten für die APBI zu analysieren.

Table of Contents

Summary 4
Zusammenfassung 5
Protocol Synopsis 9
Flow Chart 11
Abbreviations 12
1. Introduction 14
1.1 Scientific Background 14
1.2 Trial Rationale/ Justification 17
1.3 Benefit/ Risk Assessment 17
2. Trial Objectives 19
2.1 Primary Objective 19
2.2 Secondary Objectives 19
2.3 Explorative Objectives 19
3. Trial Design 20
4. Trial Duration 20
5. Selection of Patients 20
5.1 Number of Patients 20
5.2 General Criteria for Patients' Selection 20
5.3 Inclusion Criteria 20
5.4 Exclusion Criteria 21
5.5 Criteria for Withdrawal 21
5.5.1 Withdrawal of Patients 21
5.5.2 Handling of Withdrawals 21
5.5.3 Replacement of Patients 22
5.5.4 Premature withdrawal of patients from the study 22
5.5.5 Premature end of trial/ Withdrawal of the whole study 22
5.6 Prior and Concomitant Illnesses 23
5.7 Prior and Concomitant Treatments 23
6. Radiation Therapy 23
6.1 Treatment Planning for APBI and dose prescription 23
6.2 Treatment Planning for whole breast irradiation and dose prescription 23
6.3 Randomization 24
6.4 Study visits 24
7. Trial Methods 28
7.1 Assessment of Efficacy Parameters 28

7.1.1 Primary endpoint: Fatigue Testing ... 28
7.1.2 Secondary endpoints ... 28
7.1.3 Explorative Endpoint ... 32
8. Plan for Treatment or Care after the Trial ... 32
9. Assessment of Safety ... 32
9.1 Adverse Events ... 32
9.2 Serious Adverse Events ... 33
10. Statistical Considerations ... 34
10.1 Sample Size Calculation ... 34
10.2 Analysis ... 34
10.2.1 Analysis sets ... 34
10.2.2 Confirmatory analysis ... 34
10.2.3 Further analyses ... 35
11. Quality assurance ... 35
11.1 SOP (Standard operating procedures) ... 35
11.2 Data quality ... 36
12. Documentation ... 36
12.1 Data management ... 36
12.2 Patient identification list ... 37
12.3 Investigator Site File (ISF) ... 37
12.4 Data storage ... 37
13. Reports, Publications ... 37
13.1 Final Report ... 37
13.2 Publications ... 37
14. Ethical, Legal and Administrative Aspects ... 38
14.1 Responsibilities of the Principal Investigator ... 38
14.2 Ethics committee, DEGRO expert committee ... 39
14.3 Patient information and informed consent ... 39
14.4 Patient insurance ... 40
14.5 Data Protection and medical confidentiality ... 40
15. Funding ... 41
16. Amendments ... 41
17. Signatures ... 42
18. References: ... 43

Protocol Synopsis

Trial Population

Women with early stage, low risk breast cancer eligible for breast-conserving surgery

Inclusion Criteria

- Histologically confirmed invasive breast cancer
- Total tumor size < 2.5 cm
- cN0
- estrogen receptor positive, HER2-negative on immunohistochemistry
- age ≥ 50 years
- ECOG Performance status ≤ 2
- Ability of subject to understand character and individual consequences of the clinical trial
- Written informed consent (must be available before enrolment in the trial)

Exclusion Criteria

- G3
- Extensive microcalcifications
- Invasive lobular carcinoma
- Clinically involved lymph nodes
- No invasive axillary lymph node staging planned
- Patients with significant mental or physical comorbities that preclude regular follow-up
- Neoadjuvant chemotherapy or neoadjuvant endocrine therapy
- previous radiotherapy of the breast
- Known carcinoma $<$ 5 years ago (excluding Carcinoma in situ of the cervix, basal cell carcinoma, squamous cell carcinoma of the skin) requiring immediate treatment interfering with study therapy
- Pregnant or lactating women
- Participation in another competing clinical study or observation period of competing trials

Objectives

Primary Endpoint:

Between group-change in fatigue from baseline (before treatment start/surgery) compared to 12 weeks after treatment start as assessed by the FACIT Fatigue Scale questionnaire.

Secondary Endpoints:

fatigue 5 weeks, 6 months, 2 years, 5 years after treatment start

local tumor control in the index quadrant of the breast

local tumor control in the ipsilateral breast

regional tumor control

distant tumor control

overall survival

disease-free survival

secondary malignancies

acute and chronic toxicity

quality of life

cosmesis

Gene expression analysis using the Prosigna/PAM50-assay to evaluate the potential prognostic/predictive impact on choice of radiotherapy modality

Trial Design

Prospective, randomized 2-armed Phase II Trial

Sample Size

202 patients

Trial Duration and Dates

Total trial duration: 84 months
Recruitment phase: 24 months
FSI (First Subject In): January 2019
LSI (Last Subject In): January 2021
LSO (Last Subject Out) – Primary Endpoint: April 2021
LSO (Last Subject Out) – Secondary Endpoints: January 2026

Flow Chart

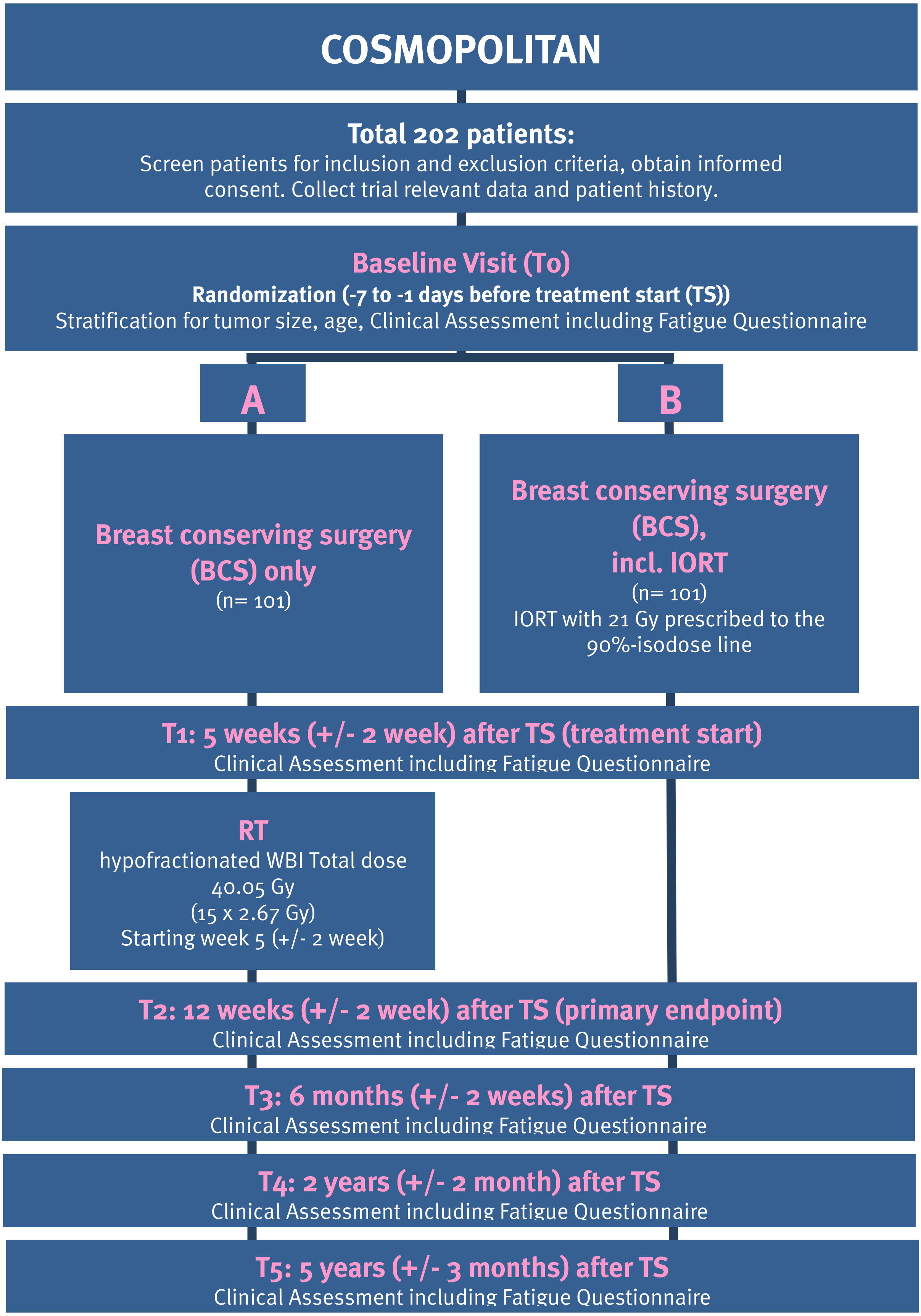
COSMOPOLITAN
Total 202 patients:
Screen patients for inclusion and exclusion criteria, obtain informed consent. Collect trial relevant data and patient history.
Baseline Visit (To)
Randomization (-7 to -1 days before treatment start (TS))
Stratification for tumor size, age, Clinical Assessment including Fatigue Questionnaire
A
B
Breast conserving surgery (BCS) only
(n= 101)
Breast conserving surgery (BCS), incl. IORT
(n= 101)
IORT with 21 Gy prescribed to the 90%-isodose line
T1: 5 weeks (+/- 2 week) after TS (treatment start)
Clinical Assessment including Fatigue Questionnaire
RT
hypofractionated WBI Total dose 40.05 Gy
(15 x 2.67 Gy)
Starting week 5 (+/- 2 week)
T2: 12 weeks (+/- 2 week) after TS (primary endpoint)
Clinical Assessment including Fatigue Questionnaire
T3: 6 months (+/- 2 weeks) after TS
Clinical Assessment including Fatigue Questionnaire
T4: 2 years (+/- 2 month) after TS
Clinical Assessment including Fatigue Questionnaire
T5: 5 years (+/- 3 months) after TS
Clinical Assessment including Fatigue Questionnaire

Abbreviations

AE = Adverse Events

AMG = German Drug Law (Deutsches Arzneimittelgesetz)

APBI = Accelerated partial breast irradiation

ASTRO = American Society for Therapeutic Radiology and Oncology

BCS = Breast conserving-surgery

BDSG = Bundesdatenschutzgesetz

BRA = Breast retraction assessment

CHT = Chemotherapy

CRF = Case Report Form

CT = Computer tomography

CTCAE = Common Toxicity Criteria for Adverse Events

CTV = Clinical target volume

DSGVO = „Datenschutzgrundverordnung“, General Data Protection Regulation

DVH = Dose volume histogram

EC = Ethics Committee

ER = Estrogen receptor

FAS= full analysis set

FDA = US Food and Drug Administration

FSI = First Subject In

GCP = Good Clinical Practice

Gy = Gray

HIRO = Heidelberger Institut für Radioonkologie

IOERT = intraoperative electron radiotherapy

IORT = intraoperative radiotherapy

ISF = Investigator Site File

ISRCTN = International Standard Randomised Controlled Trial Number

IT = Information technology

ITT = Intention-to-treat

KPS = Karnofsky Performance Score

LPI = Last Patient In

LPO = Last Patient Out

OS = Overall survival

PI = principle investigator

pBRA = Percentage BRA

PP= Per protocol

PR = Progesterone receptor

PTV = Planning target volume

QoL = Quality of Life

RCT = Randomized controlled trials

Ref = Reference length

RT = Radiation therapy

RTOG = Radiation Therapy Oncology Group

SAE = Severe adverse events

SOP = Standard operating procedures

TS = Treatment start

WBI = Whole-breast radiotherapy

1. Introduction

1.1 Scientific Background

Breast cancer is the most frequent cancerous disease in women[1, 2]. Every year, about 70 000 women are diagnosed with breast cancer in Germany[30]. The standard of care for patients with early stage breast cancer is breast conserving-surgery (BCS) followed by adjuvant radiotherapy and adjuvant systemic therapy according to the estrogen (ER) and progesterone receptor (PR) as well as human epidermal growth factor receptor 2 (HER2) status[1]. In several randomized controlled trials (RCTs) and meta-analyses, adjuvant whole-breast radiotherapy (WBI) after BCS has been shown to significantly decrease the risk of local (in-breast) recurrence and to improve breast-cancer mortality as well as overall survival[3, 4]. However, when selecting patients at a low a priori risk of local recurrence, only a small breast-cancer specific mortality benefit but no overall survival advantage was detected in the recent meta-analysis by the Early Breast Cancer Trialists Collaborative Group (EBCTCG)[4]. Consequently, several RCTs addressed the question whether the addition of adjuvant WBI to endocrine therapy improves the outcome of patients with early stage, low risk breast cancer[15-19]. However, all of the individual trials detected a significant benefit for adjuvant WBI in terms of local control while no survival benefit was found[15-19]. Matuschek et al. conducted a meta-analysis of these trials and reported a hazard ratio of 6.8 for local recurrence when adjuvant WBI was omitted[20]. Thus, adjuvant WBI remains the standard of care after BCS in patients with early stage, low risk breast cancer. Standard adjuvant WBI is currently administered as hypofractionated radiotherapy with a total dose of about 40.0 Gy in 15-16 factions for 3-5 weeks[1, 2].

As most local recurrences occur in the proximity of the primary tumor bed, partial breast-irradiation delivered exclusively to the original tumor location has gained increased attention during the past couple of years[31-33]. Hereby, smaller target volumes allow for an increase in dose per fraction combined with a decrease in overall treatment time enabling accelerated partial breast irradiation (APBI). Several different techniques have been used to deliver APBI. Multicatheter brachytherapy has been one of the first techniques studied for APBI. 10-year results are available from a small single-center RCT and show comparable outcomes after WBI and APBI with multicatheter brachytherapy[34]. In 2016, 5-year results from a multi-center RCT conducted by the GEC-ESTRO have been published showing that APBI is non-inferior compared to WBI[24].

The probably most easily implementable approach for APBI is the application of percutaneous radiotherapy with a conventional linear accelerator as it is universally available. Besides some other studies, the IMPORT LOW-Trial compared partial-breast to whole-breast radiotherapy for low-risk early breast cancer using a standard external beam technique[23]. For avoiding confounding by radiotherapy dose-time factors, the investigators kept the overall treatment time

and the total dose in the tumor bed the same in all three arms. The standard arm was hypofractionated WBI with 40.05 Gy in 15 fractions, while there was one experimental arm with a reduced dose of 36.0 Gy outside the tumor bed and another experimental arm with partial breast irradiation of 40.05 Gy only to the tumor bed. 5-year local recurrence rate were similar in all 3 arms and non-inferiority of the reduced dose-arm and the partial breast irradiation arm was shown[23]. Adverse events were comparable between the treatment arms with significantly fewer adverse effects in breast appearance and firmness in one or both of the experimental arms[23].

The ultimate form of APBI is single-dose intraoperative radiotherapy (IORT) delivered to the tumor bed during BCS. Two large RCTs employing different techniques of IORT have been published so far: the ELIOT and the TARGIT-A-trial[25, 26]. The ELIOT-trial is a single-center RCT comparing one dose of 21 Gy of electron-IORT (IOERT) to WBI with 50 Gy and a tumor bed boost over 6 weeks. Inclusion criteria were non-restrictive resulting in inclusion of a considerable number of patients with risk factors such as tumor size > 2 cm, positive nodes, G3 as well as negative immunohistochemistry for ER and/or PR[26]. 5-year results showed a local recurrence rate of 4.4% for patients receiving IORT and 0.4% for patients receiving WBI which was statistically significant, but below the pre-specified non-inferiority margin. However, when excluding patients with risk factors in the IORT-arm, the 5-year local recurrence rate was only 1.5%. Patients receiving IORT had significantly lower rates of skin toxicity and pulmonary fibrosis, but higher rates of fat necrosis.

The TARGIT-A trial is a multi-center RCT randomizing 3,451 patients to single-shot 20 Gy of 50 kV-IORT vs. 50 Gy of WBI in 25 fractions[25]. In the IORT-arm, additional WBI was permitted in the case of risk factors and was delivered in 15.2% of patients in the IORT-arm[35]. Furthermore, inclusion of patients who received IORT during a second operative procedure was allowed (post-pathology stratum). 5-year results were published in 2014, although only 1222 patients had a median follow up of 5 years. The median follow-up of the total cohort was 2 years and 5 months. The 5-year local recurrence rate was 3.3% in the IORT-arm and 1.3% in the WBI-arm (p = n.s.). Non-inferiority was demonstrated for the pre-pathology stratum, but not for the post-pathology stratum. There were significantly less non-breast cancer-related deaths in the IORT-arm, although the reason was unclear.

A meta-analysis of 5 RCTs comparing APBI to WBI was conducted by the TARGIT A-investigators[36]. While breast cancer-related mortality was similar, APBI resulted in a significant decrease of non-breast cancer-related mortality and a borderline significant improvement in overall survival when using a random-effects model[36]. Based on the above mentioned studies, both national guidelines in Germany, but also international guidelines suggest the use of APBI in patients with early stage,

low risk breast cancer[1, 2, 29, 37]. Nevertheless, most guidelines recommend the application of APBI preferably within a clinical trial[1, 29].

Adequate patient selection for APBI is crucial. While patients with early stage low risk breast cancer are believed to have non-inferior 5-year local recurrence rates following APBI compared to WBI, APBI alone is not sufficient for women with tumors showing higher risk features like larger tumor sizes, positive nodes, G3 or negative immunohistochemistry for ER and/or PR[26]. Patient selection is usually performed based on tumor stage as well as pathological and immunohistochemical markers[1, 2]. However, these markers are known to show a high inter-observer variability[38, 39]. A recent study including 3,198 breast cancer patients reported that histologic grade was discordant between central and local laboratories in 44% of the cases[38]. Another study observed substantial variability in Ki67 scoring even among some of the world's most experienced laboratories[39].

On the contrary, for facilitating the decision about the need for adjuvant chemotherapy besides endocrine treatment, current guidelines support the application of multi-gene testing for patients with early-stage hormone-receptor positive, HER2– as well as node-negative breast cancer[1, 2, 40, 41]. By using these multi-gene assays, early stage low risk breast cancer patients with a 10-year distant recurrence rate of less than 10% can be identified, who have minimal if any benefit from additional chemotherapy added to regular endocrine treatment[38, 40-43]. One of the current, well-established multi-gene assays is the PAM50-based Prosigna risk of recurrence (ROR) (NanoString Technologies) test, which has been shown to have a high prognostic relevance and was therefore approved for selecting early-stage low risk patients for whom chemotherapy and extended endocrine therapy might not be indicated[44]. As these biological markers support the classification of early-stage breast cancer patients into groups that are associated with a good or a poor prognosis, they might also be helpful for more precisely identifying early stage low risk breast cancer patients for whom APBI is sufficient instead of WBI. A recent retrospective analysis of the ABCSG-8 trial analyzing 1,308 postmenopausal endocrine responsive breast cancer patients even underlined the prognostic impact of the PAM50-assay in predicting local recurrence[45, 46].

Fatigue is one of the most common and distressing side-effects reported by breast cancer patients and occurs in more than 80% of patients during radiotherapy[5-10]. During the course of radiotherapy, fatigue usually increases, but typically subsides within weeks after the end of radiation treatment[7, 12]. However, in up to 40% of cases, it can persist long after the completion of therapy[47-49]. Fatigue is usually described as a general physical or mental exhaustion that negatively affects quality of life due to functional disability and psychological distress[11, 12]. Psychosocial problems like fatigue, depression or cognitive limitations in occupationally active

cancer survivors are distinctly associated with problems at work[50]. The potential economic impact of fatigue is considerably high: 75% of patients and 40% of caregivers are forced to change their employment status due to cancer-related fatigue[13, 14]. A recent study about employment participation in early-breast cancer patients further highlighted the enormous socioeconomic impact of fatigue by showing that fatigue patients were more likely to experience diminished employment after 2 years of follow-up[51].

Previous analyses reported that the level of fatigue rises with cumulative radiation dose and might be related to the duration of treatment[21, 52]. Both, total dose as well as field sizes are known to influence severity of fatigue[21, 22, 53]. For APBI the target volume only includes the former tumor cavity and not the whole breast tissue and total treatment time is usually reduced compared to WBI, hence early stage breast cancer patients treated with APBI instead of WBI are expected to recover faster from cancer-related fatigue leading to improved quality of life compared to patients receiving WBI.

Indeed, few first and mainly retrospective studies with small patient numbers reported lower levels of acute fatigue in breast cancer patients receiving APBI instead of WBI[22, 27, 28]. Furthermore, the GEC-ESTRO study group recently investigated quality of life following APBI with interstitial brachytherapy and described significantly reduced fatigue after APBI compared to WBI[54].

The aim of the present trial is to compare single-shot intraoperative APBI with electrons to standard hypofractionated WBI for patients with low-risk early stage breast cancer. Due to its high socio-economic and clinical impact fatigue is chosen as the primary, patient-reported endpoint for this study. The Prosigna/PAM50-assay will be applied to further evaluate the potential prognostic impact of tumor biology and gene expression-analysis in supporting adequate patient selection.

1.2 Trial Rationale/ Justification

The primary goal of this trial is to show that BCS followed by APBI with electrons results in a significantly better tolerability of adjuvant radiotherapy in terms of fatigue compared to BCS followed by hypofractionated WBRT. Fatigue is a major side effect of cancer treatments and is a main contributor to long-term quality of life-impairment as well as unemployment[11, 13, 14, 51]. The prognosis of women with early stage, low risk breast cancer is excellent and the risk of local recurrence after BCS has been shown to be in the range of 1-2% at 5 years after diagnosis[20]. Thus, maintaining a high quality of life and minimizing morbidity and mortality of adjuvant treatment is crucial.

1.3 Benefit/ Risk Assessment

At Heidelberg University Hospital, oncologic patients are treated within the National Center of Tumor Diseases (NCT), a multidisciplinary cooperation, where patients with breast cancer are

thoroughly discussed and treatment approaches are decided in tumor conferences consisting of gynecologists, oncologist, radiologists, radiation oncologists as well as pathologists. This concept assures that all patients receive the optimum treatment and are treated to the limit of professional and scientific possibilities.

In this context, the Department of Radiation Oncology performs radiotherapy in more than 740 breast cancer patients per year. The department has high experience and expertise in radiation treatments, and intraoperative radiotherapy is established as a standard therapy approach for different types of cancer since the early 1990s[55-57]. In November 2014, intraoperative single-shot electron radiotherapy was started to be applied as a boost technique for early-stage breast cancer patients therapy and since then more than 170 patients have been treated with this approach. The treatment results were recently presented at the annual meeting of the International Society of Intraoperative Radiotherapy in Mannheim, 2018 showing excellent local control combined with only mild toxicity. This well-established intraoperative radiotherapy technique will be used for APBI in the current trial.

There exists strong and prospective data supporting the hypothesis that APBI for early-stage low-risk breast cancer patients is non-inferior to WBI, the current standard of care for those patients, and that therapy-associated toxicity can even be reduced[23-26]. Those findings have already been incorporated within the respective NCCN and German guidelines[1, 2, 29]. As data regarding local control and survival following APBI is still limited to results after 5-10 years of follow-up, patients are encouraged to participate in a clinical trial[1, 2, 29]. The above mentioned and discussed ELIOT trial applied intraoperative electron radiotherapy for APBI and reported to WBI comparable local recurrence rates after excluding patients with high-risk factors in the IORT arm[58]. The American Society for Therapeutic Radiology and Oncology (ASTRO) has therefore published selection criteria for APBI which were adapted by the current NCCN guideline[37]. The ASTRO criteria will be applied for guaranteeing that only low-risk early-breast cancer patients will be treated with APBI in the current trial.

By participating in this trial, early-stage low-risk breast cancer patients have the chance to be treated with intraoperative electron APBI, an established form of treatment, which is expected to show non-inferior results for local control and survival, while deemed to be advantageous in terms of acute and chronic toxicity and dramatically shortening overall treatment time. Within this trial, the individual genetic risk profile of each patient is assessed applying the Prosigna/PAM50-assay. With the help of this multi-gene assay the decision about possibly needed further cancer-specific therapy might be facilitated. Additionally, the systematic clinical follow-up regimen administered in this trial could prove beneficial in the preservation of quality of life.

2. Trial Objectives

The purpose of this trial is to analyze intensity of fatigue in early-stage breast cancer treated with WBI or APBI after BCS. We propose that patients treated with APBI have lower fatigue levels after radiotherapy based on the FACIT Fatigue Assessment Questionnaire compared with patients treated with standard WBI.

2.1 Primary Objective

The primary objective is the assessment of fatigue from baseline (before treatment start) compared to 12 weeks after treatment start as assessed by the FACIT Fatigue Assessment Questionnaire.

2.2 Secondary Objectives

fatigue 5 weeks,6 months, 2 years, 5 years after treatment start

local tumor control in the index quadrant of the breast after 2 and 5 years

local tumor control in the ipsilateral breast after 2 and 5 years

regional tumor control after 2 and 5 years

distant tumor control after 2 and 5 years

overall survival after 2 and 5 years

disease-free survival after 2 and 5 years

secondary malignancies after 2 and 5 years

acute and chronic toxicity after 5 weeks, 12 weeks, 6 months, 2 years, 5 years after treatment start

quality of life 5 weeks, 12 weeks, 6 months, 2 years, 5 years after treatment start

cosmesis 5 weeks, 12 weeks, 2 years, 5 years after treatment start

2.3 Explorative Objectives

Gene expression analysis using the Prosigna/PAM50-assay to evaluate the potential prognostic/predictive impact on choice of radiotherapy modality

3. Trial Design

This trial will be conducted as a prospective, randomized, two-arm Phase II study.

4. Trial Duration

The overall duration of the trial is expected to be approximately 84 months. Recruitment of patients will start in January 2019. Recruitment of the patients is planned over a time period of 24 months, minimum duration of the follow-up phase will be 5 years.

Total trial duration: 84 months
Recruitment phase: 24 months
FSI (First Subject In): January 2019
LSI (Last Subject In): January 2021
LSO (Last Subject Out) – Primary Endpoint: April 2021
LSO (Last Subject Out) – Secondary Endpoints: January 2026

5. Selection of Patients

5.1 Number of Patients

202 patients will be enrolled in this phase II clinical trial.

5.2 General Criteria for Patients' Selection

Women with early stage, low risk breast cancer eligible for breast-conserving surgery will be evaluated and screened based on the protocol. All patients fulfilling the inclusion and exclusion criteria will be informed about the possibility to participate in the study. Registration for the study must be performed prior to beginning of RT.

5.3 Inclusion Criteria

Women with early stage, low risk breast cancer eligible for breast-conserving surgery meeting all of the following criteria will be considered for admission to the trial:

- Histologically confirmed invasive breast cancer
- Total tumor size < 2.5 cm
- cN0
- estrogen receptor positive, HER2-negative on immunohistochemistry
- age ≥ 50 years
- ECOG Performance status ≤ 2

- Ability of subject to understand character and individual consequences of the clinical trial
- Written informed consent (must be available before enrolment in the trial)

5.4 Exclusion Criteria

Patients presenting with any of the following criteria will not be included in the trial:

- G3
- Extensive microcalcifications
- Invasive lobular carcinoma
- Clinically involved lymph nodes
- No invasive axillary lymph node staging planned
- Patients with significant mental or physical comorbidities that preclude regular follow-up
- Neoadjuvant chemotherapy or neoadjuvant endocrine therapy
- previous radiotherapy of the breast
- Known carcinoma $<$ 5 years ago (excluding Carcinoma in situ of the cervix, basal cell carcinoma, squamous cell carcinoma of the skin) requiring immediate treatment interfering with study therapy
- Pregnant or lactating women
- Participation in another competing clinical study or observation period of competing trials

5.5 Criteria for Withdrawal

5.5.1 Withdrawal of Patients

A subject may voluntarily discontinue participation in this study at any time at their own request or at request of the legal representative. In addition, study treatment will be discontinued if unmanageable toxicity is documented, or if the Principal Investigator makes a decision to terminate the study. A subject will be withdrawn from the protocol if, in the investigator's opinion, continuation of the trial would be detrimental to the subject's well-being. If the subject withdraws from the trial and also withdraws consent for disclosure of future information, no further evaluations should be performed, and no additional data should be collected. The PI may retain and continue to use any data collected before such withdrawal of consent, in case the patient has not withdrawn the further use of his data as well.

5.5.2 Handling of Withdrawals

In all cases, the reason for withdrawal must be recorded in the Case Report Form and in the subject's medical records. In case of withdrawal of a subject at his/ her own request, the reason can be asked and documented. All efforts will be made to follow up the subjects and, all

examinations scheduled for the final trial day will be performed as far as possible on all patients and documented. All ongoing Adverse Events (AEs)/ Serious Adverse Events (SAEs) of withdrawn patients have to be followed up until no more signs and symptoms are verifiable or the subject is on stable condition.

5.5.3 Replacement of Patients

Patients will not be replaced if consent is withdrawn retrospectively and patients have already been randomized.

5.5.4 Premature withdrawal of patients from the study

Individual termination criteria during the treatment phase:

- at any time at the request of the patient
- occurrence of therapy-resistant severe side effects (CTC grade 4 toxicity, which does not recover spontaneously, after supportive therapy or after a radiation break)
- intraoperative re-classification of patient into non-low risk breast cancer, e.g. detection of positive sentinel lymph node
- R1/2 resection of the tumor

In the case of lymph node metastasis identified postoperatively, additional radiotherapy to the regional lymphatic basins should be considered. If a R1/2 resection status is diagnosed postoperatively, re-resection and additional whole-breast irradiation should be taken into account.

Individual termination criteria during the follow-up phase:

- at any time at the request of the patient

5.5.5 Premature end of trial/ Withdrawal of the whole study

Reasons for premature termination of the entire study are:

- Decision including benefit-risk assessments of the study management when unacceptable risks and toxicities occur
- Consideration of termination for each grade 5 toxicity, 2 consecutive grade 4 toxicities, 5 consecutive grade 3 toxicities
- new (scientific) evidence during the study
- Inadequate recruitment rate

5.6 Prior and Concomitant Illnesses

Relevant additional illnesses present at the time of informed consent are regarded as concomitant illnesses and will be documented in the patient chart. Abnormalities which appear for the first time or worsen (intensity, frequency) during the trial are adverse events (AEs) and must be documented on the appropriate pages of the CRF.

5.7 Prior and Concomitant Treatments

Relevant additional treatments administered to the patients on entry to the trial or at any time during the trial are regarded as concomitant treatments and must be documented on the appropriate pages of the CRF. Patients having received neoadjuvant chemotherapy or neoadjuvant endocrine therapy cannot be included in the trial. During radiation therapy, medication required for concomitant illnesses (i.e. hypertension, thyroid disease, hyperlipidemia etc.) can be applied. Concomitant medication should be discussed with the principal investigator on an individual basis. No concomitant chemotherapy or any other anti-tumor medication except for hormone therapy is allowed during the treatment period in this trial.

6. Radiation Therapy

6.1 Treatment Planning for APBI and dose prescription

Patients will receive electron IORT with a total dose of 21 Gy prescribed to the 90%-isodose. Selection of the appropriate size and shape of the tube is at the discretion of the treating physician, but a minimum diameter of 1-2 cm in addition of the clinical tumor size is suggested. The tumor bed should be prepared according to the instructions by Veronesi et al[58]. A lead or aluminum shield shall be inserted on the surface of the major pectoralis muscle to protect the thoracic wall. The linear accelerator delivers electrons at variable energies (6, 9 and 12 MeV). Depending on the target thickness the adequate electron energy is chosen.

6.2 Treatment Planning for whole breast irradiation and dose prescription

Radiotherapy is administered after full recovery from surgical resection, usually after 5 weeks of BCS. Patients will receive computed tomography-based 3-dimensional treatment planning. The total dose to the breast is 40.05 Gy in 15 fractions. The use of intensity-modulated radiotherapy and deep inspiration breath hold techniques is permitted. No tumor bed boost or regional nodal irradiation shall be delivered.

Target contouring of the CTV-breast is performed according to the ESTRO-guidelines[59, 60]. An additional margin for inter- and intrafractional positioning uncertainties of 5-10 mm is added to

generate the PTV-breast. The PTV-breast_EVAL (generated by subtraction of the 3 mm below the skin from the PTV-breast) should receive 95%-107% of the prescribed dose.

Organ at risk	Dose constraints
Heart	D_{mean} < 3 Gy, D_{max} < 30 Gy (minor violation < 40 Gy)
Ipsilateral Lung	D_{mean} < 8 Gy, V_{20Gy} < 10% (minor violation < 15%)
Contralateral Breast	D_{mean} < 3 Gy, D_{max} < 20 Gy
Spinal cord	D_{max} <45 Gy

Dose constraints of normal tissue will be respected according to QUANTEC reports (Bentzen et al. 2010; Marks et al. 2010).

6.3 Randomization

After initiation of the study, patients will consecutively be screened and eligible patients will be enrolled into the study. To achieve comparable intervention groups, patients will be allocated in a concealed fashion in a 1:1 ratio by means of randomisation using a centralised web-based tool (www.randomizer.at). Randomization will be stratified with respect to invasive tumor size (≤ 1 cm^3, > 1 cm^3) and age (≤ 60 years, > 60 years). Block randomisation with varying block lengths will be performed to achieve in total equal group sizes.

6.4 Study visits

After screening of patients for inclusion and exclusion criteria and having received informed consent, appropriate patients will be recruited to the trial. Study relevant data will be collected and patient history will be assessed. Patients will be randomized 1:1 to one of the two study arms. The baseline visit (T0) will be scheduled -7 to -1 day before planned treatment start. During the baseline visit (T0) a clinical assessment as well as analysis of cosmesis and quality of life including fatigue is planned (and FACIT Fatigue Assessment Questionnaires, see 6.4). Patients will also receive photographic documentation for cosmetic assessment.

Patients in arm A are treated with BCS followed by hypofractionated WBI with a total dose of 40.05 Gy in 2.67 Gy single doses 5 weeks (+/- 2 week) after surgery when wound healing is completed. Patients treated in arm B will receive BCS followed by intermediate single-shot IOERT to the tumor cavity with a dose of 21 Gy prescribed to the 90%-isodose line.

Both groups will be evaluated after BCS at 5 weeks after treatment start (Study Visit 1), to assess to potential influence of IORT on fatigue (secondary endpoint). In group A this time point corresponds to 5 weeks after BCS, just before the start of RT; in group B this corresponds to 5 weeks after BCS incl. IORT.

After the first study visit patients will receive regular follow-up visits based on the standard follow-up program of current German guidelines[1, 2]. The second, third, fourth and fifth study visits are planned 12 weeks (primary endpoint), 6 months, 2 and 5 years (secondary endpoints) after the treatment start. These visits will include a clinical assessment as well as analysis of quality of life including fatigue (EORTC and Fatigue Assessment Questionnaires, BREAST-Q and BCTOS, see below for details). Photographic assessment of cosmetic results is also planned except for the last visit after 5 years. The last subject in (LSI) will be followed 5 years after baseline. This is considered the final study visit.

Treatment within this trial includes in both arms therapy approaches that are standard-of-care, and hence the patients require no additional time for treatment. The follow-up visits are implemented in the clinical routine, with the exception of baseline visit 0 in group A. The clinical and quality of life assessments including detailed analysis of fatigue as well as the photographic documentation of cosmesis as part of follow-up require an additional 30 minutes approximately per visit.

- Screening –

Screening for the study will be performed prior to inclusion of the patient into the study. All inclusion and exclusion criteria must be fulfilled.

- Baseline Visit (T0)

Baseline Visit will be performed after inclusion of the patient into the study protocol. The baseline visit will be scheduled -7 to -1 day before planned treatment start/BCS.
The following examinations will be performed:

- Clinical assessment using CRF
- Quality of life using the EORTC QLQ-C30, BREAST-Q and BCTOS
- Fatigue assessment using the Fatigue Assessment Questionnaire (FACIT – Fatigue Scale)
- Photographic documentation for cosmetic assessment

- Study Visit (T1)

The first study visit is scheduled 5 weeks (+/- 2 week) after treatment start in both groups. As group B receives irradiation with intraoperative electron radiotherapy, this visit will also take place 5 weeks after RT for group B.
The following examinations will be performed:

- Clinical assessment using CRF
- Quality of life using the EORTC QLQ-C30, BREAST-Q and BCTOS
- Fatigue assessment using the Fatigue Assessment Questionnaire (FACIT – Fatigue Scale)

- Photographic documentation for cosmetic assessment
- Assessment of adverse events

-Study Visit (T2), primary endpoint

The second study visit is planned 12 weeks (+/- 2 week) after treatment start for both groups. For group A, this is 7 weeks after hypofractionated WBI, while for group B this visit is 12 weeks after IORT.

The following examinations will be performed:

- Clinical assessment using CRF
- Quality of life using the EORTC QLQ-C30, BREAST-Q and BCTOS
- Fatigue assessment using the Fatigue Assessment Questionnaire (FACIT Fatigue Scale)
- Photographic documentation for cosmetic assessment
- Assessment of adverse events

-Study Visit (T3)

The third study visit is scheduled 6 months (+/- 2 weeks) after treatment start. The following examinations will be performed:

- Clinical assessment using CRF
- Quality of life using the EORTC QLQ-C30
- Fatigue assessment using the Fatigue Assessment Questionnaire (FACIT Fatigue Scale)

-Study Visit (T4)

The fourth study visit is scheduled 2 years (+/- 2 months) after BCS. The following examinations will be performed:

- Clinical assessment using CRF
- Quality of life using the EORTC QLQ-C30, BREAST-Q and BCTOS
- Fatigue assessment using the Fatigue Assessment Questionnaire (FACIT Fatigue Scale)
- Photographic documentation for cosmetic assessment

-Study Visit (T5)

The fifth study visit is scheduled 5 years (+/- 3 months) after BCS. The following examinations will be performed:

- Clinical assessment using CRF
- Quality of life using the EORTC QLQ-C30
- Fatigue assessment using the Fatigue Assessment Questionnaire (FACIT Fatigue Scale)

Work-Flow	Baseline	Follow-up				
	To	T1	T2	T3	T4	T5
	Baseline visit after enrolment	5 weeks after TS	12 weeks after TS	6 months after TS	2 years after TS	5 years after TS
Medical history	X	X	X	X	X	X
Fatigue Assessment (FACIT Fatigue Scale)	X	X	X	X	X	X
EORTC QLQ-C30	X	X	X	X	X	X
BREAST-Q and BCTOS	X	X	X		X	
Documentation of medication	X	X	X	X	X	X
Documentation of AEs		X	X			
Photographic documentation of cosmetic assessment	X	X	X		X	
CRF Form	X	X	X	X	X	X

7. Trial Methods

The primary objective is to compare change on the overall fatigue scale from baseline (before surgery) to week 12 after treatment start between breast cancer patients treated with hypofractionated WBI or intraoperative APBI. Secondary analysis include local tumor control, regional and distant recurrence rates, disease-free as well as overall survival, assessment of acute and chronic toxicity, quality of life and cosmesis. Furthermore, gene expression analysis using the Prosigna/PAM50-assay will be performed as a secondary endpoint to evaluate the potential prognostic/predictive impact on choice of radiotherapy modality.

7.1 Assessment of Efficacy Parameters

7.1.1 Primary endpoint: Fatigue Testing

The primary endpoint is change of fatigue after APBI or WBI from baseline to 12 weeks after treatment start (T2 vs. T0). Fatigue will be assessed with the help of the Functional Assessment of Chronic Illness Therapy (FACIT) Fatigue Scale, which consists of a 13-item multidimensional self-assessment form evaluating quantity of fatigue and distress[61]. The FACIT Fatigue Scale is extensively applied in cancer patients[27, 62]. Using the FACIT Fatigue Scale questionnaire, patients are asked to indicate how frequent each item was for them "during the past 7 days" applying a 5-point scale ("not at all" to "very much"). The range of possible scores is 0–52, with 0 being the worst possible score and 52 the best.

Patient Administration time: 5-10 min

7.1.2 Secondary endpoints

Time to progression is defined as the number of days from randomization to the first occurrence of the respective event.

Fatigue

Fatigue will be assessed additionally at time points 5 weeks, 6 months, 2 years, 5 years after treatment start.

Local tumor control in the index quadrant of the breast

Local tumor control in the index quadrant of the ipsilateral breast as a secondary endpoint is defined as no tumor relapse in the breast tissue quadrant of the initial tumor/ at the site of surgical intervention. Local tumor control in the index quadrant of the ipsilateral breast is used as an additional secondary endpoint to distinguish between true local recurrences in the index quadrant from second (or new) ipsilateral carcinomas in other quadrants. In the intraoperative study arm (arm B) only the former tumor bed is irradiated, while in study arm A the whole breast tissue is irradiated.

Local tumor control in the index quadrant of the breast is taken as number of days from randomization until local tumor progression, death without prior local progression, or end of follow-up. For patients alive and not diagnosed with local progression at the end of the study, the local control time will be censored at the time of the last study visit.

Local tumor control in the ipsilateral breast

Local tumor control in the ipsilateral breast as a secondary endpoint is defined as no tumor relapse in the whole ipsilateral breast tissue. Local tumor control in the ipsilateral breast is taken as number of days from randomization until local tumor progression, death without prior local progression, or end of follow-up. For patients alive and not diagnosed with local progression at the end of the study, the local control time will be censored at the time of the last study visit.

Regional tumor control

Regional tumor control is regarded as no occurrence of regional lymph node metastases (axilla, supraclavicular fossa, internal mammary chain). Regional tumor control is defined as number of days from randomization until occurrence of regional lymph node metastases, death without prior regional tumor progression, or end of follow-up. For patients alive and not diagnosed with regional progression at the end of the study, the regional tumor control time will be censored at the time of the last study visit.

Distant tumor control

Distant tumor control is defined as no occurrence of distant metastases (lymph node metastases in the axilla, supraclavicular fossa and internal mammary chain are not classified as distant metastases, see above). Distant tumor control is defined as number of days from randomization until occurrence of distant metastases, death without prior distant progression, or end of follow-up. For patients alive and not diagnosed with distant progression at the end of the study, the distant tumor control time will be censored at the time of the last study visit.

Overall survival

Overall survival time, defined as number of days from randomization until death or end of follow-up. For patients alive at the end of the study, the overall survival time will be censored at the time of the last visit or follow-up contact.

Disease-free survival

Disease-free survival, defined as number of days from randomization until the first occurrence of local recurrence, regional lymph node metastases, distant metastases, tumor-related death, death without prior progression, or end of follow-up. For patients alive and not diagnosed with progression at the end of the study, the disease-free survival time will be censored at the time the patient was last known to be free of progression of tumor disease.

Secondary malignancies

The frequency of secondary malignancies as well as the time of their diagnosis will be assessed during follow-up visits.

Toxicity/ quality of life

Detailed acute and chronic potentially therapy-related toxicity will be assessed during each follow-up visit and documentation of side-effects with Common Terminology Criteria for Adverse Events and with the Radiation Therapy Oncology Group (RTOG)/ European Organization for Research and Treatment of Cancer (EORTC) Late Radiation Morbidity Scoring System Schema. Documentation of toxicity will mainly focus on breast shrinkage/distortion, breast induration/ fibrosis, change of skin appearance, telangiectasia, breast oedema, numbness, fat necrosis and local pain. QoL will be analyzed with the help of the validated 30-item self-assessment questionnaire of the European Organization for Research and Treatment of Cancer (EORTC QLQ-C30, version 3.0). It is composed of five multi-item functional scales (physical, role, emotional, cognitive, and social function), three multi-item symptom scales (fatigue, pain, nausea/vomiting) combined with a global health and quality-of life scale. The other six single items assess further symptoms (dyspnea, insomnia, appetite loss, constipation, diarrhea) that are often reported by cancer patients as well as financial difficulties[63]. Scores are interpreted according to the guidelines of the EORTC Scoring Manual[64].

The BREAST-Q questionnaire is a validated survey instrument especially developed for patients undergoing breast surgery. An extra BREAST-Q BCS module exists, which is specifically designed for patients undergoing BCS measuring quality-of-life (QOL) and satisfaction[65, 66]. Both of these topics are divided into three subscales i.e., QOL: physical, psychosocial and sexual well-being; Satisfaction: satisfaction with cosmetic outcome (breast appearance), satisfaction with overall outcome and satisfaction with care. Patients are asked to rate each item question on a four-point scale. The BREAST-Q is separated into a pre- and post-surgery version.

The Breast Cancer Treatment outcome scale (BCTOS-12) contains 12 items, which are assigned to two internally consistent subscales:[67] 1) Functional Status, 2) Aesthetic Status. Patients are instructed to rate each item of the BCTOS-12 on a four-point scale evaluating the differences between the treated and the untreated breast (1 = no difference, 4 = large difference). The score for each subscale is the mean of the ratings over all items belonging to that subscale. A higher score reflects a poorer status (i.e. a larger difference between the treated and the untreated breast).

Patient Administration time: 15-20 minutes

Cosmesis

Photographic assessments before and after BCS will be taken at baseline, during the first, the second and the forth study visit (T0, T1, T2, T4). Evaluation of breast cosmesis will be performed according to the proposed method by Vrieling et al[68].

Two frontal views of the chest will be taken, one with hands near the body and the other with hands raised as far as possible above the head. A profile view of the treated breast (arms above the head) is also planned for each assessment. The suprasternal notch as well as a point 25 cm below in the midline will be marked. Quantitative analysis is performed by a panel of doctors according to the graphic below. The three components of the indices (breast retraction assessment (BRA), percentage BRA (pBRA), reference length (Ref)) are determined as shown below and the resulting values are calculated:

$$BRA = \sqrt{(a_1 - b_1)^2 + (a_2 - b_2)^2} \qquad (1)$$

$$\mathrm{Ref} = \sqrt{b_1{}^2 + b_2{}^2} \qquad (2)$$

$$pBRA = \left(BRA/ref\right) \bullet 100 \qquad (3)$$

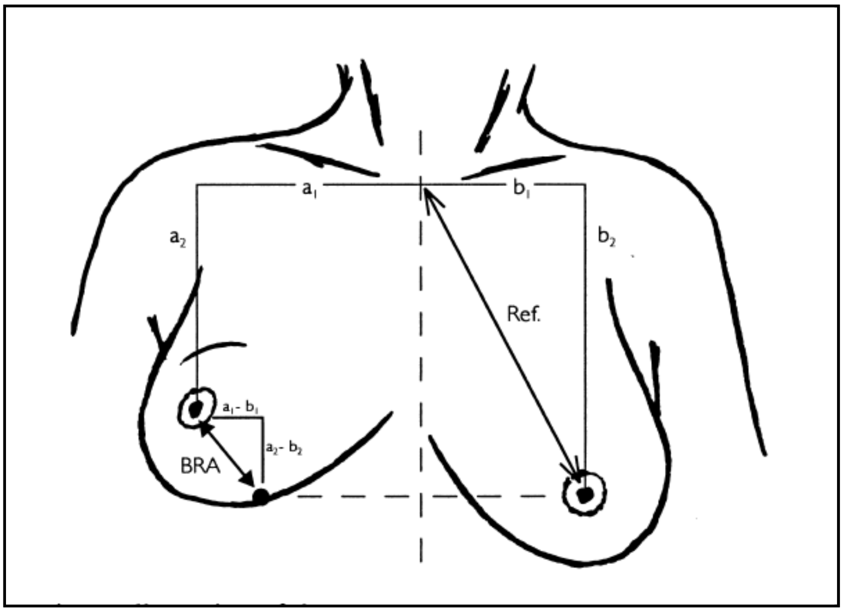

Illustration of the measurements according to Vrieling et al.[68]

7.1.3 Explorative Endpoint

Gene expression analysis using the Prosigna/PAM50-assay

The Prosigna/PAM50-assay will be applied on the operatively resected tumor tissue according to manufactor's instructions[69].

8. Plan for Treatment or Care after the Trial

After completion of study treatment, any standard treatment may be considered. Any systemic treatment or chemotherapy is not part of the clinical trial. For tumor progression, treatment alternatives will be evaluated and discussed interdisciplinary considering options of surgical resection, systemic therapy (chemotherapy, molecular targeted therapies, immunotherapy) as well as re-irradiation in certain cases.

9. Assessment of Safety

During and following a subject's participation in the trial, the investigator should ensure that adequate medical care is provided to a subject for any adverse. The investigator should inform a subject when medical care is needed for intercurrent illness(es) of which the investigator becomes aware. RT will be carried out as ambulatory treatment; however admission to the ward for supportive care is possible when necessary. Supportive measures include skin care, pain management will be performed at the discretion of the treating radiation oncologist.

9.1 Adverse Events

Toxicity associated to irradiation that occurs for the first time after treatment start or preexisting toxicity that significantly increases in the severity grade after the start of study treatment likely to be related to radiotherapy will be documented within the subject's medical records and eCRF. Treatment related side effects will be recorded according to CTCAE v5.0 (Common Terminology Criteria for Adverse Events version 5.0; http://www.eortc.be/services/doc/ctc). Grades refer to the severity of the adverse event.

Acute toxicities are defined by the occurrence within the first 90 days after the start of RT. Adverse Events occurring after the first 90 days until 6 months after study treatment will be documented as subacute toxicities. Any adverse event emerging more than 6 months after RT will be recorded as late toxicities.

Standardized informed consent forms for irradiation of breast cancers are utilized to inform the patient about study treatment. Expected acute side effects of RT include radiation dermatitis, fatigue, pain, soreness and dryness of the breast, pruritus of the skin, hairloss in the axilla, sore

throat and change in breast size, shape and color. Characteristics and severity of subacute/ late side effects depend on the location and the extent of the target volume/ irradiation field and include skin fibrosis, change in breast size, shape and color, lymphoedema, lung fibrosis, rib fractures, cardiac disorders like cardiac arrhythmia, cardiac insufficiency and coronary heart disease, soft tissue necrosis as well as brachial plexopathy.

Acute/ subacute toxicities will be assessed prior, during and after RT, including:

- Skin disorders: radiation dermatitis, dryness and pruritus of the skin, hairloss in the axilla, lymphedema
- Breast disorders: pain, swelling, change in breast size, shape and color
- Endocrine disorders: hot flashes, night sweets, bone pain
- Lung disorders: coughing, dyspnea
- Gastrointestinal disorders: nausea and vomiting
- Other disorders: alopecia, weight loss

9.2 Serious Adverse Events

A serious adverse event is an adverse event with a special degree of severity in that it

- leads to death (CTCAE grade 5)
- is life-threatening (CTCAE grade 4) due to its actual and documented severity at the point of occurrence (not only life-threatening in principle if occurrence was more severe)
- causes or prolongs hospitalization
- is a congenital anomaly/ birth defect
- leads to a lasting or otherwise significant impairment
- is an otherwise significant medical incident

The following examples are not to be considered SAE:

- Medical or surgical interventions
- Hospital admissions that are not the consequence of a medical condition (e. g. social/ convenience admissions)
- day-to-day fluctuations of pre-existing illness(es) or toxicity present or detected at the start of the study that do not worsen
- A planned hospitalization where admission did not take longer than anticipated
- Tumor progression and all medical intervention performed to offer respective therapy or relieve the symptoms of such progression

10. Statistical Considerations

10.1 Sample Size Calculation

The sample size calculation is based on the primary endpoint change of fatigue from baseline to week 12 after Treatment start (study visit T2) between the groups APBI and WBI measured via the FACT-Fatigue score. An improvement in 6 of 13 items by one point is considered clinically relevant resulting in a clinically relevant effect size of 6 points. Assuming a standard deviation of 12[70] and with a type I error rate of $\alpha = 0.05$ (two-sided), a two sample t-test requires a total sample size of n=172 patients (86 per group) to achieve a power of $1 - \beta = 0.90$ for revealing an effect of 6 points. Adjusting for the covariates baseline fatigue, age, and tumor size in a linear model is assumed to yield less unexplained variance and thus to an additionally increased power. Taking a dropout rate of 15% percent into account, n=202 patients need to be randomized. Sample size calculation was carried out using ADDPLAN v 6.1.

10.2 Analysis

10.2.1 Analysis sets

The full analysis set (FAS) includes all randomized patients and they will be analyzed according to the treatment they were randomized to.

The per-protocol population (PP) is a subset of the FAS set and compromises all patients, who were treated according to the randomized treatment as outlined in the protocol without major protocol violations.

The safety population will consist of all patients who received one of the study treatments at least once, and patients will be allocated to the treatment they actually received. This will be the primary analysis set for the safety analysis.

Each patient's allocation to the different analysis will be defined prior to the analysis. The allocation will be documented in the statistical analysis plan. During the data review, deviations from the protocol will be assessed as „minor" or „major". Major deviations from the protocol will lead to the exclusion of a patient from the PP analysis set.

10.2.2 Confirmatory analysis

The null hypothesis to be tested states that the change in the FACT-F score between baseline and week 12 after treatment start is equal for both groups:

$$H_0 : \mu_{APBI} = \mu_{WBI}$$

This hypothesis will be tested at a two-sided level of significance of $\alpha = 0.05$ against the alternative hypothesis:

$$H_1 : \mu_{APBI} \neq \mu_{WBI}$$

The hypothesis test will be conducted using a linear model with the dependent variable "change in FACT-F score between baseline and week 12 after treatment start" and the independent factors treatment group (A/B), baseline FACT-F score, tumor size, and age. The effect estimate for the treatment group will be calculated alongside a 95% confidence interval.

The confirmatory analysis will be primarily based on the FAS which is consistent with the intention-to-treat (ITT) principle by including all patients who were randomized to the two groups.

Missing data for the primary outcome variable will be replaced using multiple imputation[71] taking the covariates treatment group, baseline FACT-F score, tumor size, and age into account by application of the fully conditional specification method[72]. This will be realized using the option "FCS" of the SAS "MI" procedure which is implemented in SAS 9.4. M=10 imputations will be done and the regression approach will be used to impute the data.

10.2.3 Further analyses

In addition to the evaluation of the FAS, a PP analysis will be performed as a sensitivity analysis. The secondary outcomes will be analyzed descriptively by tabulation of the measures of the empirical distributions. According to the scale level of the variables, means, standard deviations, medians, 1st and 3rd quartiles as well as minimum and maximum or absolute and relative frequencies, respectively, will be reported. Further, for the secondary endpoints overall survival (OS), disease-free survival (DFS), local, regional, and distant tumor control, a Kaplan-Meier analysis will be performed and a between-groups comparison via a descriptive log-rank test will be conducted. Adverse and Serious Adverse Events will be tabulated and absolute and relative frequencies with 95% confidence intervals will be calculated. The severity and the relationship to the treatment will be given. Possible differences between the treatment groups will be tested using the chi-squared test. Descriptive p-values of the corresponding statistical tests comparing the treatment groups and associated 95% confidence intervals will be given. Further exploratory analyses will be performed to identify subgroups and potential moderator variables of patients profiting distinctly from the investigated interventions. Analyses will be conducted using SAS v9.4 (SAS Institute, Cary, NC)

11. Quality assurance

11.1 SOP (Standard operating procedures)

All participating investigators are guided by the study-specific SOPs that are provided by the study administration.

11.2 Data quality

To ensure data quality and consistency, internal quality control measures are carried out. For this purpose, at least 10% of all patients included up to this time (selected at random) are monitored twice a year by an internal monitor within the framework of quality assurance (internal monitoring). Monitoring includes:

- Fulfillment the inclusion criteria,
- Treatment according to the study protocol (treatment arm, medical treatment and treatment planning),
- Follow-ups according to the protocol (time points, diagnosis, evaluation of tumor size and side effects),
- Review of regulatory documentation and notification of AEs / SAEs.
- Verification of recruitment and drop-out rates.
- Verification of documentation in the HIRO (Heidelberger Institut für Radioonkologie) database
- Verification of keeping study cockpit (“Studiencockpit”) up to date and complete

After each internal monitoring, the principal investigator receives a monitoring report. This is kept in the Investigator site file (ISF).

12. Documentation

12.1 Data management

The data is collected, managed and processed electronically in the in-house HIRO research database. It is the responsibility of the principal investigator to conduct the study in accordance with applicable legal provisions and the study protocol, and that the data is entered correctly and completely in the eCRFs. All data collected in this study must be documented by authorized persons in the eCRFs. Access to the database must be authorized in writing by the principal investigator (Signature Log). Access authorization may not be passed to third parties.

Data in the HIRO database will be checked by programmed value ranges, validity and consistency checks. If necessary, queries may arise that are made using the HIRO database and authorized persons. Based on the queries, the study physician / study nurse can review and answer or correct the resulting discrepancies.

After completion of the study and after entry of all relevant data and clarification of the queries, the data base will be closed. The originals of all central study documents, including

documentation sheets, are kept at the Study Center for at least 15 years after the final report has been prepared.

12.2 Patient identification list

Study participation is recorded by registering the patient within the study cockpit ("Studiencockpit") of the clinic's hospital information system in an electronic patient identification list. It is used exclusively by the study personnel for the subsequent identification of the participating patients. This list is kept absolutely confidential and archived for at least 15 years after the end of the study. In addition, the patient's participation in this clinical trial is marked in the patient record.

12.3 Investigator Site File (ISF)

The Investigator Site File stores the documents required for the clinical study. The principal investigator is responsible for keeping the ISF up to date and complete. After completion or termination of the study, it must be kept for at least 15 years.

12.4 Data storage

The originals of all central study documents, including CRFs, are kept in the study center for at least 15 years after the final report has been prepared. The principal investigator keeps the administrative documents (correspondence with the ethics committee, study administration, study center), the patient identification list, the signed informed consent forms, copies of the CRFs and the general study documentation (protocol, amendments) for the above mentioned time. Original data of study patients (medical records) must be kept for at least 15 years.

13. Reports, Publications

13.1 Final Report

All information pertaining to this clinical trial should be treated confidentially. The statistical analysis and the preparation of a final report will be realized and signed by the principal investigator, the study coordinator and biometrician within 12 months after the closure of the database. All information contained in this report is strictly confidential.

13.2 Publications

For the international publication of the study results, this study protocol was registered in the database of the National Institute of Health (www.clinicaltrials.gov) or in the German Register of Clinical Trials (DRKS) (https://www.drks.de/drks_web/).

The results of this clinical study will be published under the responsibility of the principal investigator. The first and last authorship are reserved for the principal investigator and the study coordinator of the study if both do not wish to transfer their authorship to a third person. All information about the study must be kept confidential until then. The final publication is planned after the end of the study. The presentation of the results in the context of a publication is based on existing requirements for publications.

14. Ethical, Legal and Administrative Aspects

14.1 Responsibilities of the Principal Investigator

The principal investigator is responsible for initiating, organizing and funding the clinical trial. The clinical trial is carried out in accordance with the existing laws and regulations, in accordance with the current version of the Declaration of Helsinki and the provisions of the Radiation Protection Ordinance (Strahlenschutzverordnung) and the Radiation Protection Law (Strahlenschutzgesetz). The recommendations of Good Clinical Practice (see ICH-GCP: International Conference on Harmonization - Good Clinical Practice, in the latest version) are taken into account (http://www.ema.europa.eu/docs/en_GB/document_library/Scientific_guideline/2009/09/WC500002874.pdf).

The present study is neither a clinical trial of drugs under the German Medicines Act (Arzneimittelgesetz, AMG) nor a medical device according to the Medical Devices Act (Medizinproduktegesetz, MPG). The present study can be carried out in accordance with the requirements of § 23b MPG as a non-MPG non-AMG study ("Sonstige Studie"), since the intraoperative linear accelerator acc. §§ 6 and 10 of the MPG is entitled to carry the CE mark. The study is not aiming fort conformity assessment of the CE mark and no additional invasive or other stressful examinations are performed.

The responsibilities of the principal investigator at the study center are amongst others:

- Ensuring that all persons involved in the study are adequately informed about the study protocol, any changes to the study protocol, the treatment plan and its study-specific tasks and functions,
- ensuring that sufficient time and capacity are available to conduct the study,
- correct collection and documentation of the data,
- ensuring that the information about participants and all information is treated confidentially by all persons involved in the study,

- maintain a list of all study physicians or other suitably qualified personnel to whom essential study-specific tasks have been delegated.

14.2 Ethics committee, DEGRO expert committee

Study protocol, patient information and the informed consent form are submitted to the ethics committee of the Medical Faculty of the University of Heidelberg for professional counseling. The study will start only after receiving the approval. The Ethics Committee will be promptly informed by the principal investigator of any changes in the study protocol that may affect patient safety. In the clinical study, no ionizing radiation in humans for the purpose of medical research according to §23 StrlSchV is used, since all treatments within this protocol are clinically indicated and performed within the medical responsibility of the participating centers. An application to the Federal Office for Radiation Protection (Bundesamt für Strahlenschutz, BfS) is therefore not required. The study was submitted to the DEGRO expert committee for advice. Recruitment will not start before the committee classifies the protocol as a medical science ("Heilkunde").

14.3 Patient information and informed consent

After informing the patient- in oral and written form - of the nature, significance, implications, expected benefits and potential risks of the clinical trial, each patient must provide written informed consent to participate in the study before enrollment. The patient must be provided with sufficient time and opportunity to decide on her participation prior to the initiation of any study measures and to be able to clarify open questions with the attending physician. The informed consent form includes the date and signature of the participant and the study physician. A copy of the informed consent form and the patient information will be handed out to the participant, the original will be placed in the Investigator Site File.

Furthermore, the patient has the option to decide separately on the transfer of his data to third parties, a refusal has no effect on study participation and on the further use of his data outside the aims of this study (e.g. meta-analyses).

The study participant can withdraw the consent at any time and without stating reasons. The study participant is asked to give the reason for withdrawal, but it is pointed out that she does not have to do this. The information about the withdrawal must be documented in the patient file as well as on the participant's informed consent form. On request, a copy of the correspondingly amended informed consent form will be handed out. The treating physician/ study nurse must ensure that the revocation of consent is communicated to the data management. The datasets will remain on the in-house HIRO database and may continue to be used for scientific studies. In the case of consent withdrawel, the data may continue to be used, as long as there is no request for the complete deletion of the data.

14.4 Patient insurance

Since only clinically established therapies and diagnostics are used within the study, there is no study-specific insurance. As for treatments outside of studies, this means that study participants are not insured for the health damage or other adverse effects that they might experience in connection with participation in this study at the University Hospital Heidelberg, unless the physician or his staff meets culpable misconduct. Intent and negligence are to be regarded as culpable misconduct.

The study participants are not accident insured on the way to and from, as well as during an outpatient irradiation, with the exception of study-related appointments (i.e. TO in group A (baseline visit)).

14.5 Data Protection and medical confidentiality

The names of the patients and all other confidential information are subject to medical confidentiality and the provisions of the General Data Protection Regulation (DSGVO) as well as “Landesdatenschutzgesetz” and “Bundesdatenschutzgesetz” (LDSG or BDSG). Patient data will only be shared in pseudonymised form. Third parties do not get any insight into original documents. The prerequisite for this is the voluntary approval of the study participants in informed consent form. For this, the study participants are informed about the following:

1. Personal data collected in the context of this clinical study, in particular health and ethnic information, will be recorded in paper form and electronic in care report files (CRFs) at the radiotherapy clinic.

2. Authorized and confidential staff of the Department of Radiation Oncology and Radiotherapy, University Hospital Heidelberg can view personal data for monitoring. To ensure the quality of the study, the data may be transferred in pseudonymised form to authorized representatives of the leading study center. For this measure, the study physician is released from his medical confidentiality.

3. The collected data, including imaging material, will be exported in pseudonymised form for scientific research purposes in the field of cancer research in collaboration with other institutions (possibly with private companies or partners abroad with possibly lower data protection levels). For research purposes, the pseudonymised data may be linked to other data from other sources (such as diagnostic records, treatment planning, cancer registry, medical records, etc.).

4. The consent can be withdrawn by the patient at any time, without giving reasons and without disadvantages for further medical care. In the case of such a revocation of the consent, the

data may continue to be used, as long as there is no request for the complete deletion of the data.

5. Health data of the study participant can be collected or viewed by co-treating physicians as necessary for the proper conduct and monitoring of the study. In that regard, physicians are released from confidentiality.

6. The study participant may allow that his or her family physician / other treating physicians are informed about his or her participation in the study and may be asked for further information as part of the follow-up.

15. Funding

The trial is financed using funds of the Department of Radiation Oncology at the University Hospital of Heidelberg. According to the research agreement of our clinic with IntraOP Medical, this trial is financially supported IntraOP Medical, 570 Del Rey Avenue, Sunnyvale, CA 94085. IntraOP Medical is not involved in the design of the study, nor the collection/storage/analysis of the data gathered in this study. All persons involved (including the principal investigator and co-investigators) declare that there is no conflict of interest in connection with the implementation and evaluation of this study.

16. Amendments

In the interests of sound data analysis, changes in the study protocol are not scheduled. In exceptional cases, however, changes to the study conditions are possible. Any change to the study procedure must be made in writing, stating the reasons, and signed by all persons responsible for the study. The changes will then be considered part of the study protocol. If required (e.g., dose changes of the radiation and / or other significant changes that directly affect the safety of the study participants), the approval of the responsible ethics committees and the study participant must be obtained. Changes or additions to the study protocol can only be initiated and authorized by the principal investigator.

17. Signatures

The present trial protocol was subject to critical review and has been approved in the present version by the persons undersigned. The information contained is consistent with:

- the current risk-benefit assessment of the investigational treatment
- the moral, ethical, and scientific principles governing clinical research as set out the principles of GCP and in the applicable version of Declaration of Helsinki. The investigator will be supplied with details of any significant or new finding including AEs relating to treatment with the investigational treatment.

Prof. Dr. Dr. Jürgen Debus, Principal Investigator
Radiation Oncology

Date: Signature

____________________ ______________________________

Dr. Juliane Hörner-Rieber, Study coordinator/ Co-PI
Radiation Oncology

Date: Signature

____________________ ______________________________

Prof. Dr. Jörg Heil, Co-Investigator
Gynecology

Date: Signature

____________________ ______________________________

Dr. Johannes Krisam, Trial statistician
Institute of Medical Biometry and Informatics,

Date: Signature

____________________ ______________________________

18. References:

1. Leitlinienprogramm Onkologie (Deutsche Krebsgesellschaft DK, AWMF):. S3-Leitlinie Früherkennung, Diagnose, Therapie und Nachsorge des Mammakarzinoms. Available at http://www.leitlinienprogramm-onkologie.de/leitlinien/mammakarzinom/.
2. Liedtke C, Thill M, Jackisch C, et al. AGO Recommendations for the Diagnosis and Treatment of Patients with Early Breast Cancer: Update 2017. *Breast Care* 2017;12:172-183.
3. Clarke M, Collins R, Darby S, et al. Effects of radiotherapy and of differences in the extent of surgery for early breast cancer on local recurrence and 15-year survival: an overview of the randomised trials. *Lancet* 2005;366:2087-2106.
4. Early Breast Cancer Trialists' Collaborative G, Darby S, McGale P, et al. Effect of radiotherapy after breast-conserving surgery on 10-year recurrence and 15-year breast cancer death: meta-analysis of individual patient data for 10,801 women in 17 randomised trials. *Lancet* 2011;378:1707-1716.
5. Bower JE. Prevalence and causes of fatigue after cancer treatment: the next generation of research. *Journal of clinical oncology : official journal of the American Society of Clinical Oncology* 2005;23:8280-8282.
6. Bower JE. Cancer-related fatigue—mechanisms, risk factors, and treatments. *Nature Reviews Clinical Oncology* 2014;11:597.
7. Geinitz H, Zimmermann FB, Stoll P, et al. Fatigue, serum cytokine levels, and blood cell counts during radiotherapy of patients with breast cancer. *International journal of radiation oncology, biology, physics* 2001;51:691-698.
8. Montgomery GH, David D, Kangas M, et al. Randomized controlled trial of a cognitive-behavioral therapy plus hypnosis intervention to control fatigue in patients undergoing radiotherapy for breast cancer. *Journal of clinical oncology : official journal of the American Society of Clinical Oncology* 2014;32:557-563.
9. Potthoff K, Schmidt ME, Wiskemann J, et al. Randomized controlled trial to evaluate the effects of progressive resistance training compared to progressive muscle relaxation in breast cancer patients undergoing adjuvant radiotherapy: the BEST study. *BMC cancer* 2013;13:162.
10. Tavio M, Milan I, Tirelli U. Cancer-related fatigue (review). *International journal of oncology* 2002;21:1093-1099.
11. Berger AM, Mooney K, Alvarez-Perez A, et al. Cancer-Related Fatigue, Version 2.2015. *Journal of the National Comprehensive Cancer Network* 2015;13:1012-1039.
12. Schmidt ME, Chang-Claude J, Vrieling A, et al. Fatigue and quality of life in breast cancer survivors: temporal courses and long-term pattern. *Journal of cancer survivorship : research and practice* 2012;6:11-19.
13. Curt GA. Fatigue in cancer. *Like pain, this is a symptom that physicians can and should manage* 2001;322:1560.
14. Curt GA, Breitbart W, Cella D, et al. Impact of Cancer-Related Fatigue on the Lives of Patients: New Findings From the Fatigue Coalition. *The Oncologist* 2000;5:353-360.
15. Blamey RW, Bates T, Chetty U, et al. Radiotherapy or tamoxifen after conserving surgery for breast cancers of excellent prognosis: British Association of Surgical Oncology (BASO) II trial. *European Journal of Cancer* 2013;49:2294-2302.
16. Fyles AW, McCready DR, Manchul LA, et al. Tamoxifen with or without Breast Irradiation in Women 50 Years of Age or Older with Early Breast Cancer. *New England Journal of Medicine* 2004;351:963-970.
17. Hughes KS, Schnaper LA, Bellon JR, et al. Lumpectomy Plus Tamoxifen With or Without Irradiation in Women Age 70 Years or Older With Early Breast Cancer: Long-Term Follow-Up of CALGB 9343. *Journal of Clinical Oncology* 2013;31:2382-2387.

18. Kunkler IH, Williams LJ, Jack WJL, et al. Breast-conserving surgery with or without irradiation in women aged 65 years or older with early breast cancer (PRIME II): a randomised controlled trial. *The Lancet Oncology* 2015;16:266-273.
19. Pötter R, Gnant M, Kwasny W, et al. Lumpectomy Plus Tamoxifen or Anastrozole With or Without Whole Breast Irradiation in Women With Favorable Early Breast Cancer. *International Journal of Radiation Oncology*Biology*Physics* 2007;68:334-340.
20. Matuschek C, Bolke E, Haussmann J, et al. The benefit of adjuvant radiotherapy after breast conserving surgery in older patients with low risk breast cancer- a meta-analysis of randomized trials. *Radiation oncology* 2017;12:60.
21. Schwartz AL, Nail LM, Chen S, et al. Fatigue patterns observed in patients receiving chemotherapy and radiotherapy. *Cancer investigation* 2000;18:11-19.
22. Taunk NK, Haffty BG, Chen S, et al. Comparison of radiation-induced fatigue across 3 different radiotherapeutic methods for early stage breast cancer. *Cancer* 2011;117:4116-4124.
23. Coles CE, Griffin CL, Kirby AM, et al. Partial-breast radiotherapy after breast conservation surgery for patients with early breast cancer (UK IMPORT LOW trial): 5-year results from a multicentre, randomised, controlled, phase 3, non-inferiority trial. *Lancet* 2017;390:1048-1060.
24. Strnad V, Ott OJ, Hildebrandt G, et al. 5-year results of accelerated partial breast irradiation using sole interstitial multicatheter brachytherapy versus whole-breast irradiation with boost after breast-conserving surgery for low-risk invasive and in-situ carcinoma of the female breast: a randomised, phase 3, non-inferiority trial. *Lancet* 2016;387:229-238.
25. Vaidya JS, Joseph DJ, Tobias JS, et al. Targeted intraoperative radiotherapy versus whole breast radiotherapy for breast cancer (TARGIT-A trial): an international, prospective, randomised, non-inferiority phase 3 trial. *Lancet* 2010;376:91-102.
26. Veronesi U, Orecchia R, Maisonneuve P, et al. Intraoperative radiotherapy versus external radiotherapy for early breast cancer (ELIOT): a randomised controlled equivalence trial. *The Lancet Oncology* 2013;14:1269-1277.
27. Albuquerque K, Tell D, Lobo P, et al. Impact of partial versus whole breast radiation therapy on fatigue, perceived stress, quality of life and natural killer cell activity in women with breast cancer. *BMC cancer* 2012;12:251.
28. Perez M, Schootman M, Hall LE, et al. Accelerated partial breast irradiation compared with whole breast radiation therapy: a breast cancer cohort study measuring change in radiation side-effects severity and quality of life. *Breast cancer research and treatment* 2017;162:329-342.
29. Salerno KE. NCCN Guidelines Update: Evolving Radiation Therapy Recommendations for Breast Cancer. *Journal of the National Comprehensive Cancer Network* 2017;15:682-684.
30. (Hrsg) ZfKiRK-I. *Bericht zum Krebsgeschehen in Deutschland 2016*. Berlin: 2016.
31. Fisher B, Anderson S, Bryant J, et al. Twenty-Year Follow-up of a Randomized Trial Comparing Total Mastectomy, Lumpectomy, and Lumpectomy plus Irradiation for the Treatment of Invasive Breast Cancer. *New England Journal of Medicine* 2002;347:1233-1241.
32. Mannino M, Yarnold JR. Local relapse rates are falling after breast conserving surgery and systemic therapy for early breast cancer: can radiotherapy ever be safely withheld? *Radiotherapy and oncology : journal of the European Society for Therapeutic Radiology and Oncology* 2009;90:14-22.
33. Salvadori B, Marubini E, Miceli R, et al. Reoperation for locally recurrent breast cancer in patients previously treated with conservative surgery. *The British journal of surgery* 1999;86:84-87.

34. Polgar C, Fodor J, Major T, et al. Breast-conserving therapy with partial or whole breast irradiation: ten-year results of the Budapest randomized trial. *Radiotherapy and oncology : journal of the European Society for Therapeutic Radiology and Oncology* 2013;108:197-202.
35. Vaidya JS, Wenz F, Bulsara M, et al. Risk-adapted targeted intraoperative radiotherapy versus whole-breast radiotherapy for breast cancer: 5-year results for local control and overall survival from the TARGIT-A randomised trial. *Lancet* 2014;383:603-613.
36. Vaidya JS, Bulsara M, Wenz F, et al. Reduced Mortality With Partial-Breast Irradiation for Early Breast Cancer: A Meta-Analysis of Randomized Trials. *International Journal of Radiation Oncology*Biology*Physics* 2016;96:259-265.
37. Correa C, Harris EE, Leonardi MC, et al. Accelerated Partial Breast Irradiation: Executive summary for the update of an ASTRO Evidence-Based Consensus Statement. *Practical radiation oncology* 2017;7:73-79.
38. Gluz O, Nitz UA, Christgen M, et al. West German Study Group Phase III PlanB Trial: First Prospective Outcome Data for the 21-Gene Recurrence Score Assay and Concordance of Prognostic Markers by Central and Local Pathology Assessment. *Journal of Clinical Oncology* 2016;34:2341-2349.
39. Polley M-YC, the International Ki67 in Breast Cancer Working Group of the Breast International G, North American Breast Cancer G, et al. An International Ki67 Reproducibility Study. *JNCI: Journal of the National Cancer Institute* 2013;105:1897-1906.
40. Harris LN, Ismaila N, McShane LM, et al. Use of Biomarkers to Guide Decisions on Adjuvant Systemic Therapy for Women With Early-Stage Invasive Breast Cancer: American Society of Clinical Oncology Clinical Practice Guideline. *Journal of Clinical Oncology* 2016;34:1134-1150.
41. Krop I, Ismaila N, Andre F, et al. Use of Biomarkers to Guide Decisions on Adjuvant Systemic Therapy for Women With Early-Stage Invasive Breast Cancer: American Society of Clinical Oncology Clinical Practice Guideline Focused Update. *Journal of Clinical Oncology* 2017;35:2838-2847.
42. Cardoso F, van't Veer LJ, Bogaerts J, et al. 70-Gene Signature as an Aid to Treatment Decisions in Early-Stage Breast Cancer. *New England Journal of Medicine* 2016;375:717-729.
43. Sparano JA, Gray RJ, Makower DF, et al. Prospective Validation of a 21-Gene Expression Assay in Breast Cancer. *New England Journal of Medicine* 2015;373:2005-2014.
44. Sestak I, Buus R, Cuzick J, et al. Comparison of the Performance of 6 Prognostic Signatures for Estrogen Receptor-Positive Breast Cancer: A Secondary Analysis of a Randomized Clinical Trial. *JAMA oncology* 2018;4:545-553.
45. Fitzal F, Filipits M, Fesl C, et al. Predicting local recurrence using PAM50 in postmenopausal endocrine responsive breast cancer patients. *Journal of Clinical Oncology* 2014;32:1008-1008.
46. Gnant M, Filipits M, Greil R, et al. Predicting distant recurrence in receptor-positive breast cancer patients with limited clinicopathological risk: using the PAM50 Risk of Recurrence score in 1478 postmenopausal patients of the ABCSG-8 trial treated with adjuvant endocrine therapy alone. *Annals of oncology : official journal of the European Society for Medical Oncology* 2014;25:339-345.
47. Bower JE, Ganz PA, Desmond KA, et al. Fatigue in long-term breast carcinoma survivors: a longitudinal investigation. *Cancer* 2006;106:751-758.
48. Bower JE, Ganz PA, Desmond KA, et al. Fatigue in breast cancer survivors: occurrence, correlates, and impact on quality of life. *Journal of clinical oncology : official journal of the American Society of Clinical Oncology* 2000;18:743-753.

49. Noal S, Levy C, Hardouin A, et al. One-Year Longitudinal Study of Fatigue, Cognitive Functions, and Quality of Life After Adjuvant Radiotherapy for Breast Cancer. *International Journal of Radiation Oncology*Biology*Physics* 2011;81:795-803.
50. Duijts SF, van Egmond MP, Spelten E, et al. Physical and psychosocial problems in cancer survivors beyond return to work: a systematic review. *Psycho-oncology* 2014;23:481-492.
51. Ekenga CC, Perez M, Margenthaler JA, et al. Early-stage breast cancer and employment participation after 2 years of follow-up: A comparison with age-matched controls. *Cancer* 2018;124:2026-2035.
52. Hickok JT, Morrow GR, Roscoe JA, et al. Occurrence, Severity, and Longitudinal Course of Twelve Common Symptoms in 1129 Consecutive Patients During Radiotherapy for Cancer. *Journal of Pain and Symptom Management* 2005;30:433-442.
53. Beard CJ, Propert KJ, Rieker PP, et al. Complications after treatment with external-beam irradiation in early-stage prostate cancer patients: a prospective multiinstitutional outcomes study. *Journal of Clinical Oncology* 1997;15:223-229.
54. Schafer R, Strnad V, Polgar C, et al. Quality-of-life results for accelerated partial breast irradiation with interstitial brachytherapy versus whole-breast irradiation in early breast cancer after breast-conserving surgery (GEC-ESTRO): 5-year results of a randomised, phase 3 trial. *The Lancet Oncology* 2018;19:834-844.
55. Beer M, Eble MJ, Wannenmacher M, et al. [Intraoperative electron irradiation (IORT) of urologic tumors. Initial results of a pilot study of local recurrences of renal cell cancers]. *Der Urologe Ausg A* 1994;33:110-115.
56. Eble MJ, Herfarth C, Wannenmacher M. [Fundamentals and possibilities of intraoperative radiotherapy]. *Deutsche medizinische Wochenschrift* 1993;118:981-985.
57. Eble MJ, Quentmeier A, Ewerbeck V, et al. [Methodology, technical prerequisites and postoperative morbidity of intraoperative radiotherapy (IORT) of soft tissue sarcomas. Heidelberg Krankengut 6/91-9/92]. *Der Radiologe* 1993;33:513-519.
58. Veronesi U, Gatti G, Luini A, et al. Intraoperative radiation therapy for breast cancer: technical notes. *The breast journal* 2003;9:106-112.
59. Offersen BV, Boersma LJ, Kirkove C, et al. ESTRO consensus guideline on target volume delineation for elective radiation therapy of early stage breast cancer. *Radiotherapy and Oncology* 2015;114:3-10.
60. Offersen BV, Boersma LJ, Kirkove C, et al. ESTRO consensus guideline on target volume delineation for elective radiation therapy of early stage breast cancer, version 1.1. *Radiotherapy and Oncology* 2016;118:205-208.
61. Yellen SB, Cella DF, Webster K, et al. Measuring fatigue and other anemia-related symptoms with the Functional Assessment of Cancer Therapy (FACT) measurement system. *Journal of Pain and Symptom Management* 1997;13:63-74.
62. Cella D, Lai J-s, Chang C-H, et al. Fatigue in cancer patients compared with fatigue in the general United States population. *Cancer* 2002;94:528-538.
63. Aaronson NK, Ahmedzai S, Bergman B, et al. The European Organization for Research and Treatment of Cancer QLQ-C30: a quality-of-life instrument for use in international clinical trials in oncology. *Journal of the National Cancer Institute* 1993;85:365-376.
64. Treatment EOfRaC. EORTC QLQ-C30 Scoring Manual

Available at https://www.eortc.be/qol/files/SCManualQLQ-C30.pdf.
65. Pusic AL, Klassen AF, Scott AM, et al. Development of a New Patient-Reported Outcome Measure for Breast Surgery: The BREAST-Q. *Plastic and Reconstructive Surgery* 2009;124:345-353.

66. Pusic AL, Lemaine V, Klassen AF, et al. Patient-Reported Outcome Measures in Plastic Surgery: Use and Interpretation in Evidence-Based Medicine. *Plastic and Reconstructive Surgery* 2011;127:1361-1367.
67. Hennigs A, Heil J, Wagner A, et al. Development and psychometric validation of a shorter version of the Breast Cancer Treatment Outcome Scale (BCTOS-12). *The Breast* 2018;38:58-65.
68. Vrieling C, Collette L, Bartelink E, et al. Validation of the methods of cosmetic assessment after breast-conserving therapy in the EORTC "boost versus no boost" trial. *International Journal of Radiation Oncology • Biology • Physics* 1999;45:667-676.
69. *Prosigna™ Breast Cancer Prognostic Gene Signature Assay [Package Insert].* Seattle, WA: NanoString Technologies, Inc; 2013.
70. Moadel AB, Shah C, Wylie-Rosett J, et al. Randomized controlled trial of yoga among a multiethnic sample of breast cancer patients: effects on quality of life. *Journal of clinical oncology : official journal of the American Society of Clinical Oncology* 2007;25:4387-4395.
71. van Buuren S. *Flexible Imputation of Missing Data.* . Boca Raton: Chapman & Hall/CRC.; 2012.
72. van Buuren S. Multiple imputation of discrete and continuous data by fully conditional specification. *Statistical methods in medical research* 2007;16:219-242.
